# Effects of Early Adversity and War Trauma on Learning Under Uncertainty

**DOI:** 10.1111/desc.70049

**Published:** 2025-08-08

**Authors:** Matteo Lisi, Julia Michalek, Kristin Hadfield, Rana Dajani, Isabelle Mareschal

**Affiliations:** ^1^ Department of Psychology Royal Holloway University of London Egham UK; ^2^ Wolfson Institute of Population Health Queen Mary University of London London UK; ^3^ School of Psychology Trinity College Dublin Dublin Ireland; ^4^ Trinity Centre for Global Health Trinity College Dublin Dublin Ireland; ^5^ Biology and Biotechnology Department The Hashemite University in Zarqa Zarqa Jordan; ^6^ Centre for Brain and Behaviour Department of Psychology School of Biological and Behavioural Sciences Queen Mary University of London London UK

## Abstract

In uncertain situations, individuals rely on prior experiences of successes and failures to guide future decisions. Research has shown that children exposed to early adversity, such as abuse, can exhibit atypical behaviours in probabilistic learning tasks compared to peers without such experiences, which may have long‐term behavioural consequences. Building on these findings, our study investigates whether children exposed to war‐related trauma and forced displacement show similar alterations in decision‐making under uncertainty. We conducted a series of experiments involving tasks that required learning and decision‐making under uncertainty (e.g., multi‐armed bandits and foraging) in Amman, Jordan, comparing Syrian refugee children (ages 7–12) with age‐matched Jordanian non‐refugee peers. Although our first experiment suggested less exploratory behaviour in refugee children, results from subsequent tasks revealed a pattern better explained by heightened sensitivity to rewards. This interpretation suggests that refugee children's decision‐making is driven by a stronger response to rewards across different contexts. Such heightened reward sensitivity may influence how these children approach problem‐solving and decision‐making, potentially leading to detrimental outcomes in environments that benefit from greater exploration and the maintenance of a stable strategy.

**Summary:**

- Syrian refugee children (ages 7–12) showed heightened reward sensitivity compared to age‐matched Jordanian peers across decision‐making tasks.
- Reward sensitivity influenced children's choices under uncertainty, particularly following successful risky outcomes.
- Findings highlight how early adversity may shape decision‐making strategies relevant to resilience and long‐term cognitive development.

## 1 Introduction

Childhood exposure to adversity and trauma has been consistently linked to long‐term negative outcomes, including poor mental health, behavioural problems and suboptimal decision‐making (Black et al. 2017; Brent and Silverstein 2013). For instance, children subjected to abuse can display disruptions in associative learning (Hanson et al. 2017; Harms et al. 2018), alterations in reward processing (Birn et al. 2017) and deficits in social skills such as emotion recognition, the ability to infer emotions from facial expressions (Pollak and Kistler 2002). Such disturbances, compounded by environmental factors, can predispose these children to developmental difficulties and mental health problems (Brown et al. 2008; Nanni et al. 2012), as well as elicit detrimental behaviours such as substance addiction (Afifi et al. 2012; Elwyn and Smith 2013; Puetz and McCrory 2015), or increased aggressivity (Feerick et al. 2002).

Sadly, childhood maltreatment and neglect represent just a fraction of adversities many children face. Indeed, with the increase in conflicts around the world, a pressing global concern is adversity that relates to war exposure and displacement (UNICEF 2016). Astonishingly, more than 117 million people are estimated to have been forcibly displaced, of whom children account for a staggering 40% (UNHCR 2024). Past research in conflict zones underscores the prevalence of traumatic exposures among refugee children (Betancourt et al. 2017; Goldstein et al. 1997; Morgos et al. 2007; Panter‐Brick et al. 2009). Though precise data on the long‐term effects of such traumatic experiences is still being gathered, preliminary findings have highlighted their association with poor mental health (Fazel et al. 2012; Hodes and Vostanis 2019), academic struggles and challenges to psychosocial wellbeing (Aghajafari et al. 2020; Graham et al. 2016).

We recently examined the effects of early adversity related to war and displacement on Syrian refugee children's affective development. We found that although Syrian children did not exhibit the types of emotion recognition deficits reported in Western children who had experienced abuse and neglect, Syrian children with high trauma exposure displayed an increased attentional allocation to threat‐related stimuli (Michalek et al. 2021, 2022, Michalek et al. 2024) which may predispose, or reinforce, maladaptive behaviours. These findings underscore the importance of considering the heterogeneity of childhood adversities rather than assuming that all stressors exert identical influences on neurocognitive processes (McLaughlin et al. 2014). Indeed, as Smith and Pollak (2021a) note, categorising adversities too broadly (‛lumping’) or treating them as entirely distinct (‛splitting’) can obscure important commonalities and differences across experiences. Instead, they propose a topological approach, acknowledging that different adversities may share overlapping dimensions whilst still exerting unique effects on children's development. As such, even if children who experienced war trauma and forced displacement have intact emotion recognition capabilities, we cannot rule out a priori that they might not experience disruptions in other cognitive domains, such as those involved in executive functions.

The aim of the present study was to investigate how the effects of war trauma and forced displacement affect children's learning skills and decision‐making processes that are critical to healthy development (Pine et al. 2005; Steinberg and Morris 2001). Research has illustrated how decision‐making evolves from early childhood, with children displaying a reduction in random exploratory behaviours and an increase in model‐based exploration and inductive inference (Blanco and Sloutsky 2021; Meder et al. 2021; Schulz et al. 2019). These developmental changes are often conceptualised as balancing the exploration‐exploitation dilemma, which refers to how individuals weigh sampling new information (exploration) against leveraging known rewards (exploitation) (Sutton and Barto 2018; Wilson et al. 2014). Recent theoretical accounts propose that early adversity may shift this balance, accelerating the transition from exploration to exploitation, with potentially broad and lasting effects on cognition and behaviour (Frankenhuis and Gopnik 2023).

Interestingly, the existing literature suggests complex effects of early adversity on children's exploration‐exploitation behaviours. Hanson et al. (2017) observed heightened random exploratory behaviour in children with a history of physical abuse, suggesting that altered learning patterns could contribute to behavioural problems. Conversely, Humphries et al. (2015) reported increased exploitative behaviours among previously institutionalised children performing a balloon pumping task, interpreting this as an adaptive strategy shaped by environmental demands. Extending these findings to adult populations, Lloyd et al. (2022) found that adults with a history of childhood maltreatment tend to explore less, displaying lowered sensitivity to rewards and a tendency to underweight recent reward history. Although it is known that early‐life stress may have a profound impact on learning mechanisms and cognitive functioning beyond childhood, the exact pathways of such impairments are not yet clear. Poverty, environmental inconsistencies and heightened exposure to threat — the main signatures of childhood adversity — may create learning environments that lack the predictability and support needed for efficient cognitive development, potentially impairing basic learning processes (Harms et al. 2018). Supporting this view, early stress has been linked to both structural and functional changes in brain circuits underlying learning, attention, cognitive flexibility and reward processing (e.g., Hanson et al. 2017; Hanson et al. 2021; Lewis‐Morrarty et al. 2012). Early‐life stress also interacts with resilience, the capacity for positive outcomes in contexts of significant stress, which is fundamental to successful adolescent development. Although much of the existing resilience literature has focused on external protective factors such as caregiving, education and community support (e.g., Ager and Metzler 2017; Masten et al. 2021; Ungar and Theron 2020), the role of individual‐level cognitive processes, such as decision‐making under uncertainty, remains under‐explored. This is especially important given that adaptive decision‐making is critical for navigating complex and changing environments, and may serve as a key psychological resource supporting resilience. Although there is a growing body of research on resilience, especially among African youth (e.g., Theron et al. 2023; Somefun et al. 2023), the connection between adversity, decision‐making and resilience in children remains poorly understood. Understanding how adversity shapes children's decision‐making, a core aspect of healthy development, is therefore essential for advancing our understanding of developmental resilience.

Building on this premise, we examined whether children exposed to war and displacement would display similar alterations in decision‐making strategies. Using child‐friendly paradigms across four different decision‐making tasks, we compared Syrian refugee children's choices with those of their non‐refugee Jordanian peers. Based on previous research, we hypothesised that the instability experienced by Syrian refugee children would manifest as reduced exploration and increased exploitation. However, although our initial study, a standard associative learning task (Pessiglione et al. 2006), suggested signs of less exploratory behaviour, subsequent experiments designed to isolate exploration–exploitation from other factors (e.g., risk preference and reward sensitivity) did not replicate this finding. Instead, our evidence converged on an alternative explanation: Syrian refugee children showed heightened sensitivity to rewards (Galván 2013). Taken together, these results suggest that war trauma and forced displacement may induce nuanced changes in how children respond to rewards during uncertain decision‐making, rather than driving a straightforward shift in exploration–exploitation tendencies.

## 2 General Methods and Participant Characteristics

### 2.1 Participants

Participants across the three studies were Syrian refugee and Jordanian non‐refugee children living in Amman, Jordan, aged between 7 and 12 years old. Data collection took place in participants’ homes, community centres and schools in different Amman neighbourhoods, across five different timepoints between April 2019 and March 2020. All participating children were recruited through a collaborating NGO, *Taghyeer*. Details of the location and dates of data collection are provided in Table 1 below.

**TABLE 1:** Data collection location details.

Study	Amman neighbourhood	Testing location	*N*	Time
**1**	Sweileh	Participants’ homes	16	April 2019
	Al Hashmi Al Shamali	Shaqa'eq Al Nouman School	9	
		Awael Al Khair Community Center	6	
	Sweileh	Khadija Bint Khuwailed Community Centre	44	June 2019
	Sahab	Al Bonya School	11	
	Al Hashmi Al Shamali	Shaqa'eq Al Nouman School	2	
		Awael Al Khair Community Center	4	
**2**	Al Zohour	Zaha Culture Centre	132	October 2019
**3**	Al Zohour	Zaha Culture Centre	87^a^	March 2020

^a^Children who also participated in Study 2.

### 2.2 General Procedure and Mental Health Questionnaire Measures

The questionnaires were administered with Qualtrics (Qualtrics, Provo, UT) and all experimental tasks were run in Matlab (Mathworks) on a Dell laptop. The full list of questionnaires used in each study is provided in Table 2 below. All questionnaire measures were self‐reported by the children, apart from children's exposure to war‐related trauma (TEC, Panter‐Brick et al. 2009), which was reported by their primary caregiver. All questionnaires and computer task instructions were provided in Arabic by female fieldworkers who explained each task carefully and verified that the children understood the instructions after discussion by asking children questions about the tasks (in some cases where appropriate, practice trials were introduced). The fieldworkers administered the questionnaires by reading all measures aloud to the children and using visual scales to indicate response options. All questionnaires were either developed and validated in Arabic or had been adapted to Arabic and locally validated by previous studies. The mental health and wellbeing questionnaires showed good internal reliability measured with Cronbach's alpha (all measures between *α* = 0.91 and *α* = 0.65). The child‐reported scale measuring negative relationship with the caregivers showed only acceptable to poor reliability (between *α* = 0.70 and 0.35).

**TABLE 2:** Questionnaire measures used in studies 1–3.

		Study		
Measure name	Measured concept	1	2	3
Children's Revised Impact of Events Scale (CRIES‐8, Perrin et al. 2005)	Symptoms of PTSD	*α* = 0.91	*α* = 0.87	*α* = 0.82
Arab Youth Mental Health Scale (AYMHS, Mahfoud et al. 2011)	Symptoms of anxiety and depression	*α* = 0.83	*α* = 0.88	*α* = 0.75
Human Insecurity and Distress Scale (HIDS, Ziadni et al. 2011)	Feelings of insecurity	*α* = 0.79	*α* = 0.84	*α* = 0.76
	Feelings of distress	*α* = 0.77	*α* = 0.87	*α* = 0.80
Youth Life Orientation Test (YLOT, Ey et al. 2005)	Optimism	*α* = 0.65		
Psychological Control—Disrespect Scale (PCDS, Barber et al. 2012)	Negative mother‐child relationship	*α* = 0.59	*α* = 0.65	*α* = 0.39
	Negative father‐child relationship	*α* = 0.70	*α* = 0.60	*α* = 0.35
Traumatic Events Checklist^a^ (TEC, Panter‐Brick et al. 2009)	Exposure to traumatic events related to war and displacement (caregiver‐reported)	*x*	*x*	*x*

*Note: α* = Cronbach's alpha reliability estimate, *x* = measure collected in that study (*α* not applicable).

^a^Trauma checklist reported by the caregiver.

### 2.3 Ethics

All studies received ethical approval from the Queen Mary University of London research ethics board (QMERC2018/54 and QMERC22.115). Caregivers gave their informed written consent and children their verbal assent prior to taking part in the studies. A collaborating fieldworker explained the studies to the children, emphasising that they could withdraw at any time. Depending on the study, families were reimbursed either 4 or 10 Jordanian dinars (JOD) for travel expenses, and children received small gifts, such as stickers and sweets, as a thank‐you for participating.

## 3 Study 1: Valence‐Dependent Learning

### 3.1 Participants

A total of 92 children (47 girls) participated in Study 1, equally split between Syrian refugees and Jordanian non‐refugees, all living in Amman, Jordan. Data collection was conducted in participants’ homes, schools and community centres, in Amman in April (*n* = 31) and June (*n* = 61) 2019 (see Table 1 for details). Due to external issues and impediments that occurred during data collection, two Jordanian non‐refugee children did not complete the experiment. We included them in the modelling analysis since our multilevel modelling approach allows us to include the smaller datasets obtained from these children in a statistically principled way; however, they were excluded from other analyses that assume completion of the experiment, such as the comparison of the final scores. We also could not collect survey data from four Syrian refugee children; however, their behavioural data were still included in the modelling analysis. No formal power analysis was performed. Data collection for Study 1 (and subsequent studies) was conducted alongside our previous work on attention and emotion recognition (Michalek et al. 2021, 2022, Michalek et al. 2024). In planning, we ensured that resources would allow for a sample size at least as large as in comparable developmental studies using similar protocols (Hanson et al. 2017; Manning et al. 2017). Ultimately, we enrolled as many participants as feasibility allowed during fieldwork.

### 3.2 Procedure

**FIGURE 1:**
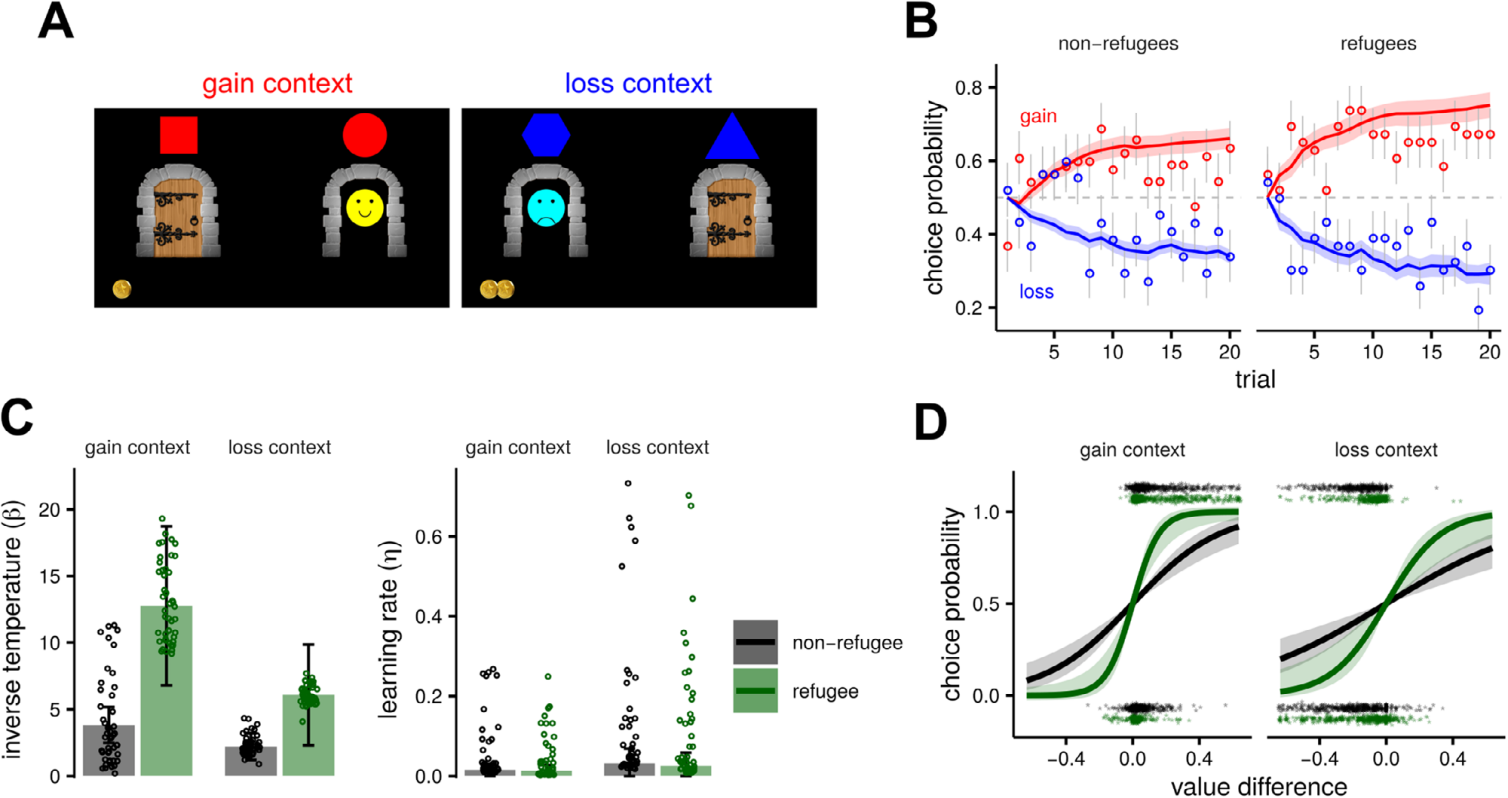
Valence‐dependent learning. (A) Monster learning task with distinct gain and loss contexts. Red symbols above the doors (left) indicated the gain context where children could find the ‘good monster’ (the yellow smiley face), who would give them one golden coin. Conversely, blue symbols indicated a loss context and the possibility of losing one golden coin if they chose the door with the ‘bad monster’ (the blue sad face). To make the outcome of the trial evident, whenever the child won or lost a coin, it was moved in a randomly generated smooth trajectory (generated as a Bézier curve) that connected the bottom of the screen (where all the gained coins would line up) and the monster. (B) Choice probabilities as a function of trial number, split by group (refugees and non‐refugees) and trial context. Data points indicate the average choice frequency for each trial (error bars represent binomial standard errors); lines and shaded bands are the predictions of the reinforcement learning model with standard errors across children. (C) Estimated parameters of the reinforcement learning model, dots indicate parameters of individual children, whereas the columns with error bars indicate posterior means of the fixed‐effects with the associated standard errors (i.e., the standard deviation of their marginal posterior distribution). The estimated values indicate that whilst refugee and non‐refugee children did not differ in their learning rates, refugee children exhibited less variable and more exploitative choices. This can also be seen in panel **D**, which shows the inferred choice functions (giving choice probability as a function of the difference in value between the two options). The shaded bands are the standard error of the predicted probability and the dots above and below the functions represent the value difference in each trial (as estimated by the reinforcement learning model).

Children performed a 2‐armed bandit task with potential gains and losses. The task required that on every trial, the child chose to open one of two virtual ‘doors’ displayed on a computer screen (Figure 1A). Children were told that one of two ‘monsters’ would be hiding behind one of the doors. There was a good monster (a yellow, happy smiley face), who rewarded children with one golden ‘coin’, and there was a bad monster (a blue, sad face), who would steal one of their previously earned coins. The children's task was to collect as many golden coins as possible by finding the good monster and avoiding the bad one. In order to make explicit the probabilistic nature of the task, children were told that each monster had a preference for hiding behind one of the two doors, as indicated by a specific symbol above the door, but the monster occasionally hid behind the other door. Each door was distinguished by one of four different geometric shapes (2 shapes × 2 colours), with the colour specifying the valence of the trial: red symbols would indicate a gain context (i.e., there was a good monster hiding behind one of the two doors, and the child could win a coin) and blue symbols the loss context (i.e., there was a bad monster hiding behind one of the doors and the child could lose a coin). On any given trial, both doors had the same colour, thus defining the valence of the trial as either a ‘potential win’ trial or a ‘potential loss’ trial. Within the pair of doors of the same valence, one door had a symbol associated with a high‐probability outcome (80% chance that the monster was hiding behind the door) and the other door a symbol associated with a low‐probability outcome (20% chance of the monster hiding behind the door). The child's task was to learn which symbol was associated with which probability. After explaining the task to the children and answering any questions, each child started with 10 practice trials. If they understood the task, this was then followed by 40 experimental trials. If they did not understand the task, we repeated the practice, explaining the outcome on each trial. The pairings between symbols and probability were randomly set and could differ between practice and experimental trials; that is to say, children were told that the monster's preference for a particular symbol could be different in the experiment compared to the practice. A subset of 34 children (12 Syrian refugees and 18 Jordanian non‐refugees) ran a longer version of the experiment, completing 60 trials in total. As mentioned above, the experiment was interrupted half‐way after only 11 and 18 trials for 2 non‐refugee children, however, they were also included in the modelling, as the multilevel modelling approach used here can naturally handle imbalanced data (participants with fewer trials carry less information about group‐level estimates and are weighted less in the multilevel model).

### 3.3 Analysis

The data were analysed by means of a standard reinforcement learning model (See Supporting Material for details). The model was fit at the group level using a hierarchical Bayesian approach (which estimates parameters for each child individually whilst also pooling information across all children). We fitted the model by using Hamiltonian Monte Carlo (HMC) sampling as implemented in Stan and its R interface (Carpenter et al. 2017; R Core Team 2024) to estimate the posterior distribution of the parameters. Between‐groups comparisons unrelated to the reinforcement learning model's parameters, were conducted with two‐tailed *t*‐tests. Bayes factors for the *t*‐tests were calculated using the JZS prior (Rouder et al. 2009). Questionnaire scores instead were analysed with non‐parametric tests, as the scores are ordinal (i.e., they can be ranked, but the differences between scores are not meaningful, as they are not measured on an interval scale). Unless stated otherwise, standard errors of the mean (SEM) throughout the paper were calculated via 10^4^ bootstrap iterations using the bias‐corrected‐and‐accelerated method (Efron 1987).

### 3.4 Results

#### 3.4.1 Demographics

There was no difference in the mean age of the two groups (Syrian refugees’ mean age: 9.17 [SD 1.84] years old and Jordanian non‐refugees mean age: 9.59 [SD 1.68] years old), *t*(86) = 1.12, *p* = 0.27. The Bayes factor (null/alternative) indicates that the data were 3.43 times more likely under the null hypothesis. The proportion of girls also did not differ significantly between the two groups (Syrian refugees: 0.37, Jordanian non‐refugees: 0.57), χ^2^(1) = 1.58, *p* = 0.21. The Bayes factor (null/alternative) was 7.67. We also asked the children how many people lived in their household and how many bedrooms were in their houses, and took the number of bedrooms per person as a proxy of socioeconomic status, which is a commonly used metric (Cable and Sacker 2019; Krieger et al. 1997). Although it does not include all aspects of socioeconomic status, either persons‐per‐room or persons‐per‐bedroom are valid proxy measures (Cable and Sacker 2019) used in many studies across many countries (e.g., Aldridge et al. 2021; Chhetri 2023; Duarte Neves et al. 2024; Samms‐Vaughan and Lambert 2017; Zaneva et al. 2024). This value was found to be similar across the two groups (Syrian refugees: 0.41, Jordanian non‐refugees: 0.40), *t*(85) = 0.41, *p* = 0.68; with the Bayes factor (null/alternative) of 5.63. Therefore, overall, the children in the two groups were well‐matched in terms of basic demographic characteristics (see Supporting Material for more details on proxy measures of socioeconomic status).

#### 3.4.2 Questionnaires

To examine any differences in trauma exposure and mental health between the refugee and non‐refugee children, we conducted non‐parametric comparisons between their questionnaire scores. We found significant differences in the caregiver‐reported trauma, which was significantly higher in the Syrian refugee group (*M* = 6.37) compared to the Jordanian non‐refugee group (*M* = 0.43), with a Bonferroni corrected *p* value of 5.91 × 10^−11^. Additionally, child‐reported PTSD was also significantly higher in the refugee (*M* = 11.74) than the non‐refugee groups (*M* = 3.05), at a corrected *p* value of 1.19 × 10^−4^. There were no significant differences between the refugee and non‐refugee groups on all the other measures of mental health and wellbeing (Table S3).

#### 3.4.3 Model‐Free Analyses

On average, Syrian refugee children ended the experiment with more coins than non‐refugee children. Refugees had 7.3 coins (SEM 0.6), and non‐refugees had 5.5 coins (SEM 0.45), *t*(88) = 2.4, *p* = 0.02. This result points to a difference in how children in the two groups approached the task. To uncover information about the underlying mechanism, we analysed the data using a mechanistic reinforcement learning model (see Supporting Material for details).

#### 3.4.4 Modelling Results

The estimated group‐level parameters are reported in Table S1. Overall, the model provides a qualitatively good fit to the children's choices (Figure 1B). The results reveal that both groups of children had slightly higher learning rates and choice variability (lower inverse temperature parameter) in the loss context, as compared to the gain context (Figure 1C), mirroring the results of previous studies in adults that used a similar protocol (Pessiglione et al. 2006). In reinforcement‐learning terms, a learning rate indicates how strongly someone updates their beliefs about an option's value after each outcome; a higher learning rate means beliefs shift more dramatically when new feedback appears. When comparing the two groups, we found a higher inverse‐temperature parameter for refugee children, for both gain and loss contexts, although the 95% Bayesian credible interval did not include zero (see Table S1) for the gain context only. The ‛inverse temperature’ parameter reflects how consistently an individual acts on what they have learned. A lower inverse temperature means more unpredictable, exploratory choices; a higher inverse temperature means more consistent, ‘greedy’ use of current knowledge. Thus, the modelling analyses suggest that refugees and non‐refugees did not differ in how they updated their beliefs about each door's expected value, but rather they differed in how systematically they chose the door with higher subjective value. This difference can be visualised in Figure 1D, where the predicted probability of choosing the door with higher reward probability is plotted as a function of the subjective value difference as estimated by the reinforcement learning model. The logistic functions are steeper for Syrian refugees, indicating that for a given difference in subjective value, they were more likely to choose the higher‐value door than Jordanian non‐refugees.

One possible interpretation of the pattern that emerged from the modelling of Study 1 is that non‐refugee children adopted a more exploratory strategy. That is, they prioritised gathering information, whereas refugees were more ‘exploitative’ in their choices and prioritised immediate rewards. However, providing a stronger test of this explore/exploit hypothesis in children of this age is challenging, because directed (i.e., uncertainty‐driven) exploration in these probabilistic tasks seems to emerge only later during adolescence, whereas younger children seem to use more random exploration (picking alternatives in a seemingly haphazard way) rather than deliberately seeking new information (Somerville et al. 2017; Nussenbaum and Hartley 2019). In the context of multi‐armed bandit tasks, it is difficult to disentangle random exploration from other factors such as motivation. To sidestep this issue, we designed Study 2 to implement a virtual foraging decision task, where participants are asked to decide between staying with a current known option or searching the environment for an unknown, potentially better alternative (Constantino and Daw 2015). This task has been shown to reveal over‐exploitation in conditions of both acute physiological and chronic subjective stress in adults (Lenow et al. 2017).

## 4 Study 2: Explore/Exploit Decisions in a Virtual Foraging Task

### 4.1 Participants

A total of 132 children (*M* age = 9.56 [SD = 17.4], 54 females) participated in Study 2, 52 of whom were Syrian refugees. No statistical methods were used to pre‐determine sample size.

### 4.2 Procedure

**FIGURE 2:**
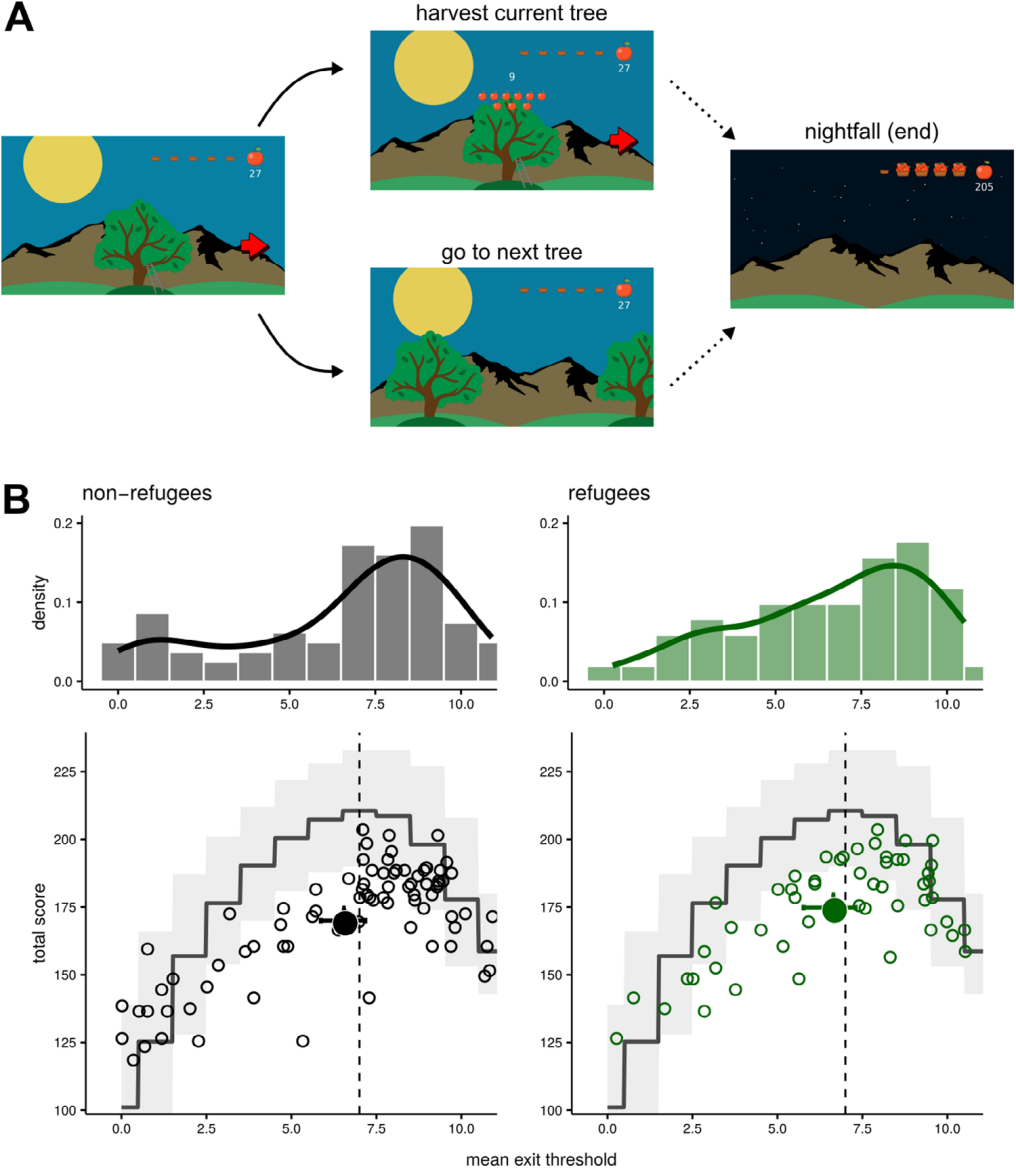
Virtual foraging task (Study 2). (A) In each trial, children decide whether to continue harvesting apples from the current tree by clicking on it or to switch to a new tree by clicking on a red arrow. Each decision (stay or move) advances the sun downward in the background; when the sun sets behind the mountain, the game ends. (B) The lower panels show each child's total score (vertical axis) plotted against their exit threshold (horizontal axis) — the minimum number of apples at which they switched trees. Small empty dots represent individual children; filled dots represent the group mean. The dark grey line indicates the expected total score for each exit threshold if it were followed consistently throughout the game, and the light grey band shows the 95% prediction interval. The dashed vertical line is the optimal exit threshold (7 apples). Both Jordanian (left) and Syrian (right) groups displayed average thresholds near the optimal value. The upper panels show the distribution of exit thresholds.

The task was based on Constantino and Daw's (2015) foraging task and consists of a series of decisions between harvesting a currently selected tree for apples (’harvest' decisions) or moving to a new tree (‛exit’ decisions; Figure 2A). Each tree's initial supply of apples is drawn from a normal distribution with a mean of 10 and a standard deviation of 1 (rounded to return an integer number of apples). The first harvest yields a number of apples identical to the tree's initial supply; each subsequent harvest yields a number of apples corresponding to the previous harvest multiplied by a decay factor and then rounded. The decay factor was drawn independently for each harvest from a Beta distribution with parameters *α* = 14.9 and *β* = 2, chosen such that the average depletion rate was 0.88 with a standard deviation of 0.07 (Lenow et al. 2017). These settings create a broad range of trees that differ in terms of the total supply of apples. To make this more child‐friendly, the main difference from the previous implementation of this task (Constantino and Daw 2015; Lenow et al. 2017) was that we discretised time elapsed by a finite number of turns or choices (rather than providing a fixed amount of time for children to do the task). This was necessary to minimise the impact of interruptions or distractions (due to the testing conditions, where multiple children were tested simultaneously in the same classroom), ensuring that incidental pauses would not affect performance. To make the passage of time explicit, we modified the display to include a cartoon background with the sun setting behind two mountains (Figure 2A). More specifically, with each choice (either to harvest or to move to the next tree), the sun moved a step downwards and the sky became darker. Children were told that they had to collect as many apples as possible before sunset, which occurred after 30 choices. To enhance the gamification of the task and highlight the progress in the number of apples collected, we added a set of empty baskets to the display, which would get filled one by one after every 50 apples collected. The task was explained to the children by local fieldworkers who discussed it with them and answered any questions to ensure they understood the procedure before starting. Each child was then asked to restate what they needed to do, confirming that they understood the procedure before beginning. Their performance supports this understanding, as their average choices aligned closely with the task's optimal threshold for exit decisions (see Results).

### 4.3 Analysis

Following Constantino and Daw (2015), we calculated each child's mean *exit threshold*: the minimum number of apples obtained from a harvest before an exit decision. This was calculated as the average number of apples returned from the last two harvests before an exit decision was made. These thresholds were compared to the optimum threshold for the task, which we derived by numerical simulations (Figure 2B). Additionally, we examined trial‐by‐trial choices using a logistic model in which the probability of harvesting the current tree was a function of the expected number of apples (see Supporting Material for details). The model was fit at the group level using a hierarchical Bayesian approach. We also tested a variant of this model that included a lapse parameter controlling the probability of ‘random’ responses (i.e., independent of the apples received).

Note that although the best strategy to approach this task is to keep track of the rewards (number of apples) obtained with each harvest, a simpler alternative strategy could be to switch to a new tree after a fixed number of harvests—essentially ignoring the size of the rewards. To verify that children paid attention to the rewards and used them to guide their choices, we fit an alternative model that implemented this alternative strategy. The model was similar to the logistic regression model mentioned above, with the difference that choice probability was taken to be a logistic function of the preceding number of harvests collected in the same tree rather than the expected number of apples at the next choice (see Supporting Material for details). We compared the model based on preceding rewards against the model based on the preceding number of harvests using the WAIC (Widely Applicable Information Criterion) criterion (Vehtari et al. 2017), a statistical measure that balances how accurately a model explains the data against how complex the model is.

### 4.4 Results

#### 4.4.1 Demographics

There was no difference between the mean ages of the two groups (Syrian refugees 9.39 [SD 1.69] years, Jordanian non‐refugees 9.67 [SD 1.72]) *t*(130) = 0.90, *p* = 0.37; the Bayes factor (null/alternative) was 4.93. The proportion of females also did not differ significantly (non‐refugees 0.41, refugees 0.41), χ^2^(1) = 0.00, *p* = 1; the Bayes factor (null/alternative) was 9.07. The number of bedrooms per person was also similar across the two groups (non‐refugees 0.41, refugees 0.39), *t*(130) = 0.82, *p* = 0.41; the Bayes factor (null/alternative) was 5.23. We also compared the two groups based on the proportion of currently employed people per household, which was also similar across the two groups (non‐refugees 0.24, refugees 0.21), *t*(130) = 1.17, *p* = 0.24; the Bayes factor (null/alternative) was 3.76. Overall, this suggests that children in the two groups were well‐matched across demographics characteristics and socioeconomic status.

#### 4.4.2 Questionnaires

We conducted the Wilcoxon rank‐sum test to establish if refugee children differed from non‐refugee children in their trauma exposure and mental health outcomes. We found that refugee children were exposed to significantly more traumatic events (*M* = 6.71) compared to non‐refugee children (*M* = 1.55) with the Bonferroni corrected *p* value of < 0.001. All other self‐reported mental health measures did not differ between refugee and non‐refugee children (see Table S5).

#### 4.4.3 Virtual Foraging Task

There was no difference in the estimated exit thresholds between the groups (refugees 6.65 [SD = 2.72], non‐refugees 6.54 [SD = 3.09]), *t*(130) = 0.19, *p* = 0.85). The Bayes factor (null/alternative) was 7.10. When analysing trial‐by‐trial choices, we found that the model that implemented a strategy based on the rewards (i.e., the expected number of apples at the next harvest) provided a better fit than the alternative model based on the preceding number of apples harvested. The model comparison revealed that the model based on rewards provided a better fit to the data (see Supporting Material, section 2.1). This confirms that children performed the task as expected and complied with the instructions, paying attention to the rewards and using them to guide their choices. Furthermore, the model also performed much better than the one augmented with a lapse rate parameter, which was the worst‐fitting model among the ones tested. We also examined whether participants’ exit thresholds deviated from the optimal threshold of seven apples. Although both groups’ averages were slightly below 7, the deviations were not significant (all *p* > 0.18), and the Bayes factors indicated moderate evidence in favour of the null hypothesis (i.e., no reliable deviation from the optimal threshold; the Bayes factor was 6.00 for refugee children and 4.83 for non‐refugee children).

Importantly, the winning model's estimated parameters were nearly identical for both groups (Table S4), indicating that children used similar foraging strategies. By design, this virtual‐foraging paradigm requires participants to compare the current reward with the expected average reward available elsewhere, capturing a form of the exploration–exploitation dilemma (Constantino and Daw 2015). Within this framework, neither group showed a stronger tendency to leave (explore) or remain (exploit) than the other, providing no support for the idea of reduced exploration among refugees. Consequently, we reconsidered whether the more exploitative pattern observed in Study 1 might instead stem from other factors, such as risk avoidance or heightened reward sensitivity. To distinguish between these possibilities, we designed Study 3 to include a ‛safe’ option (a door that always yielded a smaller but certain reward). If the reduced variability in Study 1 was driven by a desire to avoid risk, refugee children would be more likely to pick this guaranteed option; if it arose from stronger motivation for rewards, they would be drawn to the larger — but uncertain — payoff, especially following a successful gamble.

## 5 Study 3: Differential Weighting of Certain and Uncertain Rewards

### 5.1 Participants

In this study, we recruited children from the same community centre as in Study 2, all of whom had previously participated in Study 2. Data collection took place in March 2020, just before the first Covid‐19 lockdowns in European and Middle Eastern countries. Although at the time restrictions to mobility and lockdowns had not yet been enforced in Jordan, most schools had already implemented cautionary measures and paused all extra‐curricular activities. This reduced the number of children available to participate in our study. Although we aimed to collect a sample size equivalent to that of Study 2, we were only able to collect data from a total of 87 children (43 females), 35 of whom were Syrian refugees. This cohort of children ranged in age between 7 and 13 years old (*M* age = 9.83, SD = 1.80).

### 5.2 Procedure

**FIGURE 3:**
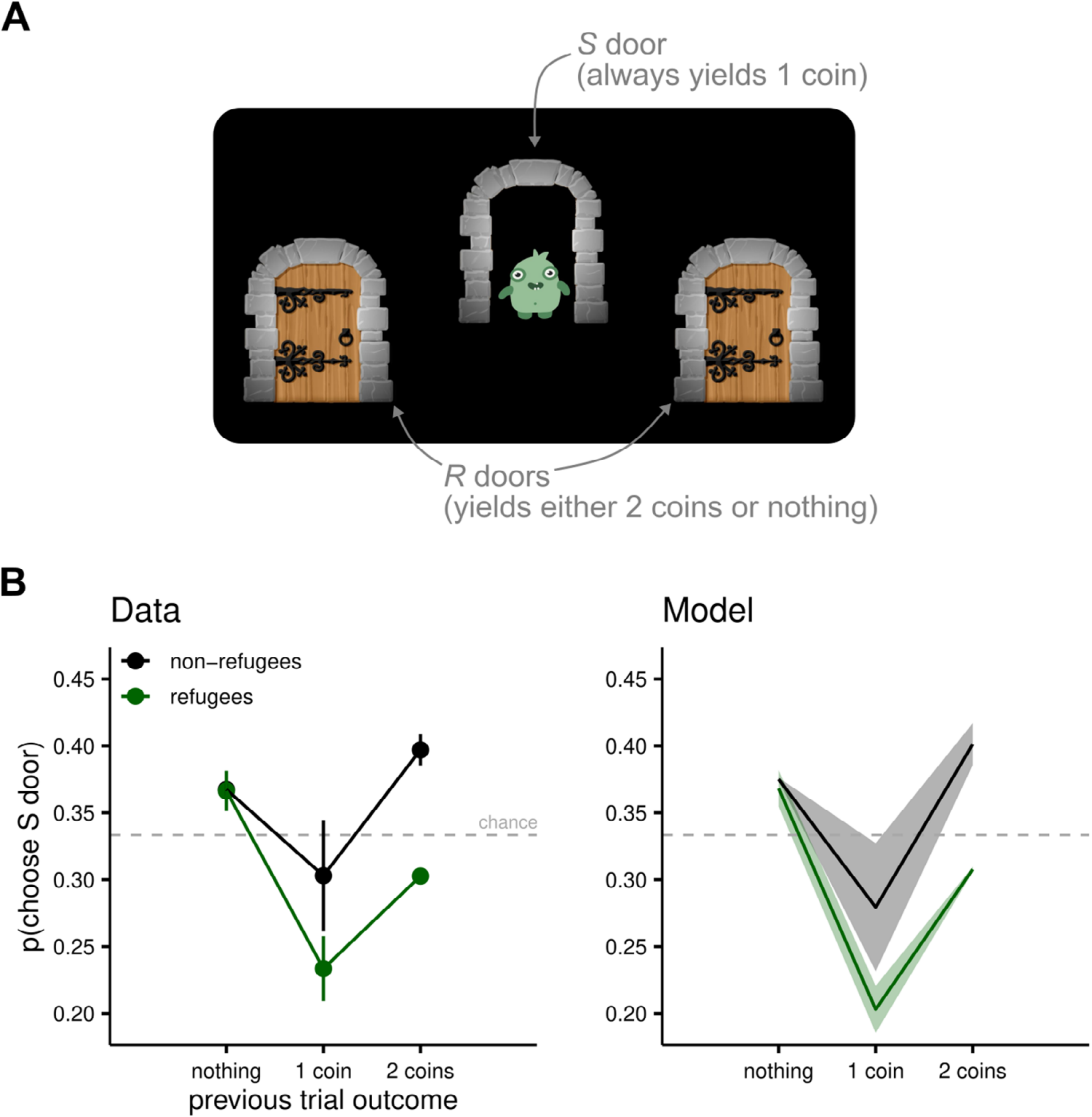
Weighting certain and uncertain rewards (Study 3). The task was designed as a multi‐armed bandit problem, in which children had to decide on every trial which door to open to maximise their rewards. (A) Example of display with the central ‘sure’ (*S*) door open. The monster (shown in the open door) rewards children with two coins; however, the monster has a relatively low probability of appearance (see Methods for details), and thus, if children choose the *R* doors, they risk not obtaining any coins. (B) Data (left) and model predictions (right). Each plot illustrates the probability of choosing the *S* doors as a function of the reward obtained in the previous trial. Outcomes of zero or two coins result from choosing an *R* door, and outcomes of one coin result from choosing the *S* door. In all panels, horizontal dashed lines indicate the proportion of choices that would be obtained if children were randomly choosing each door with the same frequency. Note that the data shows a tendency to alternate *R* and *S* choices: after choosing an *S* door, children are more likely to choose one of the *R* doors, and vice versa. Additionally, after choosing an *R* door, the next choice is influenced by the reward obtained only for refugee children (green line and dots): differently from non‐refugees, refugee children are more likely to persist with an *R* door after winning a reward of two coins. Instead, after choosing an *R* door and not winning anything, both groups are more likely to choose the *S* door than by chance. This pattern is fully accounted for only by the best‐fitting model (see Supporting Material for details).

The experimental protocol was based on Study 1 with some important differences. First, trials could only have a positive valence (i.e., it was not possible to lose coins). Second, on every trial, there were three doors, arranged horizontally with the central door positioned slightly higher than the other two on the screen. The central door represented the ‘sure’ (*S*) option and when it was chosen, this door always revealed the same monster that always gave the child one coin (Figure 3B). The other two doors were the ‘risky’ options (*R*), which could reveal a second monster that gave the children two coins (Figure 3A). The two *R* doors had different reward probabilities: one of the two revealed the monster 65% of the time, whereas the second one 35% of the time (left vs. right balanced across children). The probabilities in this task were designed such that choosing randomly one of the two *R* doors has the same expected value as continuously choosing the *S* door. However, as the experiment progresses, children gather more information about which of the two *R* doors is associated with a higher reward probability, hence increasing the expected value of *R* choices above that of the *S* door. Based on the structure of the experiment, we expected that children would choose the *S* door more frequently at the beginning of the experiment than at the end. We ensured children understood the instructions by the fieldworkers discussing the task with them and answering questions. Each child then began with 10 practice trials, and if they felt they understood the task, this was followed by 30 experimental trials; otherwise, the practice was repeated. The association between reward probabilities and left/right *R* doors was selected randomly and independently for practice and experimental trials. As in Experiment 1, coins would move over a smooth, randomly generated path from the monster to the bottom of the screen, where they accumulated over the course of the experiment along a horizontal line. At the end of the experiment, each child was rewarded with stickers and a sweet.

### 5.3 Analysis

We examined children's performance in this task by comparing it to 4 alternative computational models (see Supporting Material, section 3). All models were fit using a hierarchical Bayesian approach and were implemented in Stan (Carpenter et al. 2017). Formal comparison between the four models was performed using the WAIC (Widely Applicable Information Criterion) criterion (Vehtari et al. 2017). The four models were:

‐ *Naive value learning model*. This model ‛learns’ the value of all doors (including the guaranteed safe door, ‛S’) from scratch, ignoring the fact that the safe door has a known payout.
‐ *Value learning model*. Similar to the naive model, but here the safe door's value is not learned from experience. Instead, it is treated as a fixed, estimated constant that does not change over time.
‐ *Dual value learning model*. This model separates the learning process for the ‛risky’ (R) doors from the decision of whether to pick a risky door or the guaranteed safe door. It assumes the child might be using two parallel processes: (a) learning which R door is better and (b) deciding whether a risky choice is worthwhile compared to the safe door.
‐ *Reward‐dependent stickiness model*. This model starts with the value learning approach but adds ‛stickiness’ parameters, which capture a child's tendency to repeat a choice if it was recently rewarded. In other words, if a child was successful with one door on the last trial, they are more likely to pick that door again—independent of what they have learned about each door's value.

For detailed descriptions of these models, please see the Supporting Material.

### 5.4 Results

#### 5.4.1 Demographics

There was no difference in the mean age of the refugee and non‐refugee groups (mean age refugees 10.08 [SD = 1.82] years, non‐refugees 10.56 (SD = 1.85]), *t*(85) = 1.17, *p* = 0.24. The Bayes factor (null/alternative) was 3.16. The proportion of females also did not differ: non‐refugees 0.54, refugees 0.43, χ^2^(1) = 0.62, *p* = 0.43; the Bayes factor (null/alternative) was 7.50.

#### 5.4.2 Questionnaires

Using the Wilcoxon signed rank test we found that within this cohort, refugee children also experienced significantly more traumatic events (*M* = 6.39) compared to non‐refugee children (*M* = 1.58, Bonferroni corrected *p* < 0.001), but there were no significant group differences in any other questionnaire measures (Table S10).

#### 5.4.3 Model‐Free Analyses

The final score (number of coins) was 31.5 (SD 4.9) for non‐refugees and 29.6 (SD 5.5) for refugees. The difference was not statistically significant, *t*(85) = 1.69, *p* = 0.09; the Bayes factor (null/alternative) was 1.61, indicating that the data did not provide reliable evidence in favour of, or against, the null hypothesis. We next analysed whether the proportion of *S* choices differed across the two groups using logistic regression. We found that refugee children made significantly fewer *S* choices; the odds of refugees choosing the *S* door were 0.80±0.08 times that of non‐refugees, *z* = 2.66, *p* = 0.008.

#### 5.4.4 Modelling Results

In line with the approach described above, we tested how well each of the four computational models fit children's choices. One observation was that children in both groups tended to alternate *S* and *R* choices, a finding that could not be explained by either the naive value‐learning model, or the value‐learning model (See Supporting Material). Indeed, given that the *S* door always yields a small reward (1 coin) the probability of choosing it again cannot decrease after choosing it in the previous trial. The latter two models (dual value learning and learning with reward‐dependent stickiness) instead include a mechanism to arbitrate explicitly between risky choices (both *R* doors) and sure choices (the *S* door). These models include a stickiness parameter that is often included in modelling of human learning to capture the tendency to repeat (or alternate) choices independently of the outcome (Gershman 2016) and which seems to be a crucial ingredient to accurately account for children's choices in this task. The dual value learning model implements a learning process specifically for comparing *S* and *R* choices, which accumulate information over trials. Although this model represents a marked improvement from the first two considered, it does not fully capture the slightly different choice patterns exhibited by refugee and non‐refugee children. As can be seen in Figure 3B, refugee children tend to choose *S* less frequently than chance (the horizontal dashed line in the figure), and to choose *R* more frequently, after a successful (2 coins) *R* choice. This pattern is not seen in non‐refugee children, who display increased frequency of *S* choices after *R* choices, regardless of the outcome, and vice versa after *S* choices.

This difference between refugees and non‐refugees was fully captured by the model with reward‐dependent stickiness (see Figure 3C), which also performed best in the model comparison (see Supporting Material). This model includes a term that modulates the propensity towards *R* choices based only on the immediately preceding trial outcome, which was significantly higher in the refugee group. The estimated parameters of this model are reported in the Supporting Material. Overall, the modelling analysis revealed that refugee and non‐refugee children differed specifically in the extent to which the most recent outcome influenced their choice strategy. Although refugee children were systematically more likely to repeat risky (R) choices after an outcome of two coins, and less likely to do so after an outcome of zero coins, non‐refugee children did not show this tendency and were more stable in their choices (i.e., less swayed by the fluctuations in the stochastic rewards). These findings support the notion that the observed group difference reflects a heightened sensitivity to rewards in refugee children.

## 6 Discussion

Using three different tasks designed to investigate decision‐making processes, we compared performance between Syrian refugee children and age‐matched Jordanian non‐refugee children (age range 6–11) living in Amman Jordan. In Study 1, we used a standard two‐armed bandit task where children could earn or lose coins depending on which door they chose (Pessiglione et al. 2006). The experimental protocol was designed to measure children's ability to learn about the likelihood of rewarding choices (gains) and punishments (losses). In line with our initial expectation, we found that refugee children displayed a tendency towards making less variable choices relative to the non‐refugee children, which suggested reduced exploratory behaviour. However, when we tested this interpretation further in our subsequent studies, we uncovered a more consistent explanation in terms of heightened reward sensitivity.

We designed Study 2 with the aim to further test the hypothesis of reduced exploration in refugee children in the context of a virtual foraging task (Constantino and Daw 2015), which has been shown in adults to reveal tendencies towards over‐exploitation in acute and chronic stress (Lenow et al. 2017). Contrary to our expectations, we found no difference in refugee and non‐refugee children's foraging behaviours, prompting us to reconsider whether the initial interpretation of reduced exploration in Study 1 might instead be explained by another mechanism. To investigate this further, in Study 3 we used a modified two‐armed bandit task that included a riskless (safe) option (i.e., a door that *always* provided a smaller but certain reward). This experiment was aimed at characterising further refugee children's behaviour and investigate whether differences observed in Study 1 could have stemmed to other factors such as avoidance of risk. By introducing a guaranteed but smaller payoff, we could observe how children weighed the certainty of a modest reward against the possibility of a larger payoff with some risk. If the differences observed in Study 1 indeed reflected a propensity to avoid risk, we would expect refugee children to favour the safe option. Conversely, if heightened reward sensitivity was driving their decision‐making, the risky option could be more appealing, particularly after a prior success. We found that refugee children chose the riskless option less frequently than their non‐refugee peers. This difference was explained by a difference in how rewards influenced subsequent choices. Specifically, we found that although non‐refugee children maintained a more stable strategy—regardless of the rewards obtained—the choices of refugee children were systematically influenced by whether or not they had received a reward in the previous risky choice. Taken together, these findings point to alterations in sensitivity to rewards whilst learning under uncertainty in refugee children. Indeed, a re‐analysis of data from Study 1 revealed that the differences observed between refugees and non‐refugees could be fully explained as differences in sensitivity to reward (see Supporting Material, section 1.2). Overall, our results suggest that refugee children who have experienced early adversity have a heightened sensitivity to rewards.

We sought to test the hypothesis of heightened reward sensitivity in refugee children more directly by conducting a fourth, pre‐registered experiment using a perceptual decision‐making task with unequal frequency of rewards between correct responses for different stimuli, an approach that has been used previously to probe alterations of reward systems (Huys et al. 2013; Pizzagalli et al. 2005). Unfortunately, due also to restrictions following the COVID pandemic, we were unable to reach the pre‐registered sample size for this experiment. Although the results of this fourth experiment show a numerical trend of increased sensitivity to reward in Syrian children, the 95% Bayesian credible interval around this difference was broad and encompassed zero. The experiment's results are thus inconclusive, and the observed trend could be attributable to chance. Nonetheless, we have included the methods and results in the Supporting Material (section 4) for completeness and transparency.

In the context of Study 1, where reward probabilities remained stable over time, the more exploitative choices of the refugee children resulted in a higher number of coins on average by the end of the experiment. Although their strategy was more optimal in this fixed‐probability task, it is important to recognise that, in real‐world scenarios, reward contingencies are usually volatile rather than fixed. Prioritising immediate rewards over strategic exploration in such environments can be detrimental to long‐term outcomes. As outlined above, we interpret the observed behaviour in Study 1 as reflecting heightened motivation in refugee children to obtain rewards, rather than merely different approaches to the explore‐exploit trade‐off. This interpretation aligns with the concept of reward sensitivity in the developmental literature (Galván 2013). Although reward sensitivity can mimic the effects of a ‛learning rate’ parameter in simple instrumental learning tasks like that of Study 1, we propose that it functions as a higher‐level or ‛hyper‐parameter’ (Frankenhuis and Gopnik 2023) that is set before learning begins and shapes the broader approach to seeking rewards. Rather than being adjusted during learning (as the learning rate would be in volatile environments), this higher‐level parameter would set overarching behavioural priorities, influencing the choice strategy (e.g., favouring exploitation over exploration). From this perspective, reward sensitivity is not confined to processing immediate rewards; it shapes decision‐making by considering both the behavioural and social context as well as future reward opportunities. This conceptualisation can also explain the findings in Study 3, where success in a previous risky choice led refugee children to engage in more frequent risky choices in future trials — a pattern not observed in non‐refugee children. This heightened reward sensitivity, whilst adaptive in some contexts, may also be detrimental in environments that benefit from more exploration and the maintenance of a stable strategy, potentially leading to suboptimal long‐term decision‐making outcomes.

One avenue for future research is to more directly evaluate how environmental unpredictability, children's subjective sense of safety and social support may shape or accentuate this heightened reward sensitivity. In contexts of real or perceived resource scarcity, prioritising immediate rewards can provide short‐term advantages that might be adaptive; however, in more stable environments, a bias towards short‐term gains may hinder the development of effective long‐term strategies. This tension reflects a key insight from developmental models of resilience, which emphasise that behaviours shaped by early adversity can serve as adaptive responses in one context but may become maladaptive in another (Ungar 2024). From this perspective, the reward‐focused decision‐making we observed in refugee children may represent a functional adjustment to unpredictable environments but could limit flexibility or long‐term planning in more stable settings. Future research should explore how these patterns interact with protective factors such as secure caregiving relationships or community supports, which may buffer potential costs and support more adaptive developmental outcomes across diverse environments.

Although collectively our results do not provide support for refugee children being less explorative than non‐refugee children, a result we expected based on the literature, it is worth noting a few important differences with previous work. Firstly, more of the previous research examining children's explore/exploit behaviours have compared children who have suffered abuse or neglect with healthy children (e.g., Hanson et al. 2017); however, there is evidence that the effects of adversity experienced by refugee children are substantially different from the effects experienced in abuse or neglect, with emerging work suggesting that some cognitive‐affective processing mechanisms, such as emotion recognition and attention allocation do not seem to differ between refugees and non‐refugees (Michalek et al. 2022; Michalek et al. 2024), whereas such processes are consistently shown to be affected by childhood maltreatment (e.g., Pollak and Kistler 2002). It is also worth highlighting that more recent evidence suggests that simply counting the number of adverse events may not fully capture the differences between children's early experiences. For example, Chen et al. (2019) found that poverty, rather than adversity, was a better predictor of executive function, which is required to perform the decision‐making tasks we investigated. We did not find evidence for differences in proxy measures of poverty in our groups (see Supporting Material), and it is possible that this may underlie our lack of group differences in explore/exploit behaviours in Study 2. More recently, Xu et al. (2023) found that some aspects of environmental uncertainty (as reflected by perceived unpredictability of caregiving and environments) is associated with reduced exploratory behaviours. We did not measure environmental uncertainty, but it is likely that children across our two groups may not have differed on this measure. Taken together our results add to a growing body of literature that suggests that children's current living conditions may be a better predictor of their cognitive behaviours (including affective and learning) than past (or indirect) experiences of adversity.

Our findings stand in contrast to studies showing that early‐life adversity leads to reduced sensitivity to rewards (see Smith et al. 2025, for a recent review). Notably, some investigations have found the opposite pattern, reporting increased reward sensitivity following some forms of early adversity (e.g., Hendrikse et al. 2022; Yang et al. 2021; Kiyar et al. 2021; Davies et al. 2022). This variability in results likely reflects the heterogeneity of adverse experiences, which can yield different developmental outcomes. Many previous studies have relied on the Adverse Childhood Experiences (ACE) framework (Felitti et al. 1998; Anda et al. 2006), which encompasses a broad range of adversities such as psychological, physical, and sexual; and typically groups individuals based on the presence of four or more adversities, creating a broad categorisation that may not capture important nuances. In contrast, our study focuses specifically on trauma related to war and displacement experienced by children who have fled war and are now living as refugees, a scenario distinct from those typically studied in ACE research.

It may not be helpful to place all types of adverse experiences under a single umbrella when examining the consequences of early adversity. This perspective aligns with dimensional models of early adversity, which suggest that different types of adversity can have distinct effects on neurobiological and cognitive systems (Berman et al. 2022). However, the current debate about the utility of dimensional models highlights that these categories can often be too vague to be useful. Smith and Pollak (2021a) propose a ‛topological’ approach as an alternative, emphasising the importance of how events are experienced by the child rather than the specific type of event. This approach suggests that it is the child's experience and the various influencing factors that shape the outcomes, not just the categorical nature of the adversity. Specifically, children's perception of safety and environmental stability may play a key role in how adversity affects their psychosocial functioning, which highlights the importance of positive social relationships as prominent factors in healthy emotional and cognitive development (Smith and Pollak 2021b; Stock et al. 2025).

Our study contributes to this debate by highlighting the need to differentiate between types of early adversity. Unlike many studies that focus on children from WEIRD (Western, Educated, Industrialised, Rich, Democratic) countries with a history of emotional abuse or neglect from caretakers, our study examines the impact of war trauma and forced displacement. This unique context provides valuable insights into how this different type of adversity can lead to different developmental outcomes. We provide evidence that war trauma, a type of adversity not usually studied in WEIRD participant samples, is associated with heightened sensitivity to rewards, contrary to the reduced sensitivity often observed in other forms of early adversity (Lloyd et al. 2022). Whether one subscribes to strictly dimensional or ‛topological’ models, our data underscore the necessity of differentiating aspects of early adversity, as different types can produce opposite outcomes. With about 47.2 million children worldwide forcibly displaced due to conflict and violence (UNICEF 2024), our findings emphasise the importance of considering the unique context of war and displacement when evaluating the effects of trauma on development. These results also offer potential guidelines for the development of interventions aimed at improving outcomes in refugees. Indeed, whilst we recently showed that a positive psychology intervention could improve refugee children's mental health (Foka et al. 2021), our findings here point to the need to embed exercises that promote cognitive and executive functions to help children develop their decision‐making skills. Such exercises might give children practice in pausing before acting and considering outcomes over a longer time horizon. This, in turn, could moderate the heightened reward sensitivity we observed and support cognitive flexibility — the ability to adapt behaviour to changing circumstances — and thereby promote better choices in everyday situations where patience and foresight are advantageous. Cognitive flexibility is associated with better outcomes on a range of different tasks (e.g., Engel de Abreu et al. 2014; Genet and Siemer 2011; Chen et al. 2014) and could serve as an important target for interventions aimed at refugee children.

It is important to acknowledge several limitations of our study. First, the testing conditions were not always fully controlled. Children were tested in community centres or schools, often in environments where other children were present, which may have led to distractions. Although this might have introduced some noise into the data, the participants' performance on the tasks was consistent with previously reported results. This suggests that any distractions likely did not significantly impact the overall findings. Moreover, our data replicated key qualitative aspects observed in adult studies conducted in WEIRD countries, such as the higher learning rate for loss compared to gain contexts in Study 1. This provides initial evidence that these protocols, typically used with Western participants, are also appropriate for use in Middle Eastern (Syrian refugee and Jordanian) children. Another consideration is the age of the refugee children (7–12 years) at the time of testing. Many were either quite young when their families left Syria or were born in Jordan, which means they may have little direct memory of the war or their time in Syria. In this context, trauma exposure was assessed based on reports from primary caregivers. Although this approach provides valuable insights, it may not fully capture the child's subjective experience of adversity, which is increasingly recognised as crucial to understanding trauma's impact (Danese and Widom 2023). This raises the possibility that caregivers' reports might not fully reflect the children's personal trauma exposure, especially given the growing emphasis on individual perceptions of adversity. A third consideration is that, whilst the Syrian and Jordanian children we tested shared many lifestyle similarities (e.g., living in same neighbourhoods, going to the same schools, attending the same Mosques, etc.) there may be subtle cultural and contextual differences in their approaches to reward and risk‐taking may have influenced their decision‐making behaviours. Further research would be needed to examine the role of culture in shaping these patterns. Finally, a potential limitation specific to Study 2 lies in the discretised nature of the foraging paradigm, which replaced the continuous ‛time cost’ from the original task (Constantino and Daw 2015; Lenow et al. 2017) with a finite number of turns. Although this modification made the task more suitable for child participants in busy testing environments, it may complicate direct comparisons to prior work that employed continuous time. Indeed, whereas Xu et al. (2023) found that children under‐explored relative to the optimal threshold in a continuous‐time foraging paradigm, our participants did not deviate significantly from the optimal exit threshold, suggesting that children in our discrete‐turn version did not systematically under‐explore. Despite these differences, our participants’ exit thresholds were broadly comparable to those reported in adult samples (Constantino and Daw 2015; Lenow et al. 2017), suggesting that this adaptation remains a credible way of capturing the range of exploration–exploitation behaviours in this task. However, further research is necessary to determine whether both versions measure exactly the same underlying processes and whether findings from continuous‐time designs — such as the relation between stress and over‐exploitation (Lenow et al. 2017) — fully generalise to the discrete‐turn version.

Taken together, our findings highlight the unique effects of war trauma exposure and experiences of forced displacement on child decision making, suggesting potentially heightened reward sensitivity in refugee children. Further work should investigate environmental uncertainty as well as children's own perceptions of adversity and safety to gain a more nuanced view of the impact that traumatic experiences may have on cognitive development. Our results also speak to developmental models of resilience, which emphasise that whilst behavioural adaptations to adversity —such as a focus on immediate rewards — may be adaptive in some contexts, they could become less advantageous in safer or more stable environments (Ungar 2024). Understanding how these decision‐making patterns interact with protective factors will be essential for identifying pathways that support positive developmental outcomes in children affected by conflict and displacement.

## Conflicts of Interest

The authors declare no conflicts of interest.

## Supporting information




**Supporting File 1**: desc70049‐sup‐0001‐SuppMat.pdf

## Data Availability

The data and code that support the findings of this study are openly available in the OSF repository https://osf.io/t5cuf/.
