# Supplementary material for: Effects of Early Adversity and War Trauma on Learning Under Uncertainty
Source: Dev Sci. 2025 Aug 8;28(5):e70049. doi: 10.1111/desc.70049 (PMC12332969; doi:10.1111/desc.70049)
Supplement: Supplementary file 1 — Supporting File 1: desc70049‐sup‐0001‐SuppMat.pdf [file DESC-28-e70049-s001.pdf]

Supplemental Material for *'Effects of early adversity and war trauma on learning under uncertainty'*

Matteo Lisi[*,†] Julia Michalek[‡] Kristin Hadfield[§,¶] Rana Dajani[‖]
Isabelle Mareschal[**]

Contents

1 Study 1: Valence-dependent learning **2**

1.1 Value learning model 2

1.2 Alternative model (value learning with altered reward-sensitivity) 4

1.2.1 Alternative model's fit to data 5

1.3 Model comparison 5

1.4 Questionnaires 6

1.5 Correlations of questionnaire scores with model parameters 7

2 Study 2: Explore/exploit decisions in a virtual foraging task **8**

2.1 Model estimation & comparison 9

2.2 Questionnaires 11

2.3 Correlations of questionnaire scores with model parameters 12

3 Study 3: Comparing certain and uncertain rewards **13**

3.1 *Naive* value learning model 15

3.2 Value-learning (with fixed value for S door) 18

3.3 Dual value learning model 20

3.4 Value learning with reward-dependent stickiness 23

[*]Department of Psychology, Royal Holloway, University of London, UK
[†]matteo.lisi@rhul.ac.uk
[‡]Wolfson Institute of Population Health, Queen Mary University of London, UK
[§]School of Psychology, Trinity College Dublin, Ireland
[¶]Trinity Centre for Global Health, School of Medicine, Trinity College Dublin, Ireland
[‖]Biology and Biotechnology Department, Hashemite University in Zarqa, Jordan
[**]Centre for Brain and Behaviour, Department of Psychology, School of Biological and Behavioural Sciences, Queen Mary University of London, UK

3.5 Model comparison 26
3.6 Questionnaires 27
3.7 Correlations of questionnaire scores with model parameters 28

4 Study 4: Reward sensitivity using a perceptual decision-making task 29
4.1 Methods 29
4.2 Analysis 31
4.3 Results 32

5 Proxy measures of socioeconomic status 36

References 37

1 Study 1: Valence-dependent learning

1.1 Value learning model

The data were analyzed by means of a standard reinforcement learning model, with separate parameters for positive/negative feedback. In brief, the model assumes that on every trial t the child makes a choice $c_t \in \{1, 2\}$ and obtains a "reward" $r_t \in \{-1, 0, 1\}$. The child maintains and updates their estimate of the value (that is the expected, long-run, reward) of each choice option - the so-called Q-values. The Q-values are updated according to

$$Q_{t+1}(c) = Q_t(c) + \eta\, \delta_t$$

where η is the learning rate. The updating is done separately for positive and negative feedbacks with possibly different learning rates η^+ and η^-. δ_t is the reward prediction error, calculated as

$$\delta_t = r_t - Q_t(c)$$

A logistic sigmoid (softmax) function is used to transform values into the probability of choosing the 'best' door, i.e. the door with maximal expected gain (or minimum loss)

$$P(c_t = 1) = \frac{1}{1 + e^{-\beta[Q_t(1) - Q_t(2)]}}$$

where β is an "inverse temperature" parameter that controls the randomness of the choices and thus the exploration-exploitation trade-off.

In total, the model had 4 free parameters, η^+, η^-, β^+, β^-. The model was fit at the group level using a hierarchical (mixed-effects) Bayesian approach, where within each group the subject-specific coefficients are assumed to have a multivariate Gaussian distribution, that is:

$$\begin{bmatrix} \Phi^{-1}\left(\eta_i^+\right) \\ \Phi^{-1}\left(\eta_i^-\right) \\ \beta_i^+ \\ \beta_i^- \end{bmatrix} \sim \mathcal{N}(\mu, \Sigma)$$

where μ is the vector of the group-level, fixed-effect coefficients and Σ a fully parametrized variance-covariance matrix. Φ^{-1} denotes the probit function, that is the inverse of the cumulative distribution function of the standard normal distribution, used to ensure that the learning rate parameters are constrained within 0 and 1. The model included 4 additional fixed-effects parameters, representing the between-group difference (on probit scale) for the learning rates and for the inverse-temperature parameter (the latter coded as deviation from unity for the between-group ratio). The fixed-effects parameters were given weakly informative Gaussian priors based on previous studies that used similar methods[2]. The parameters coding for group differences were given regularizing zero-centred Gaussian priors with standard deviation set to 2; as such the priors for the differences are agnostic to the direction of the effects but introduce into the model some skepticism toward large effect sizes. Standard deviations of random effects were given a half-Cauchy prior with scale parameter set to 1, and the covariance matrix a LKJ prior[3] with shape parameter set to 2.

We fitted the model by using Hamiltonian Monte Carlo (HMC) sampling as implemented in Stan and its R interface[4] to estimate the posterior distribution of the parameters. We run 4 chains

of 4000 samples each (2000 burn-in); convergence was assessed by verifying that there were no divergent transitions and that $\hat{R} \approx 1$ for all parameters[5]. The estimates of group-level parameters are summarized in Table 1.

Table 1: Group-level parameter. Estimates are the means of posterior distribution and intervals are Bayesian HPDI credible interval.

	Non-refugees			Refugees			Group differences		
parameter	estimate	SE	95% CI	estimate	SE	95% CI	difference	diff. SE	diff. 95% CI
η^+	0.01	0.01	(0, 0.03)	0.01	0.01	(0, 0.03)	0.00	0.02	(-0.02, 0.02)
η^-	0.03	0.05	(0, 0.06)	0.03	0.04	(0, 0.07)	0.00	0.05	(-0.06, 0.07)
β^+	3.78	1.37	(1.65, 5.9)	12.42	5.91	(4.13, 21.03)	8.64	5.14	(1, 15.6)
β^-	2.18	1.05	(0.56, 3.72)	9.32	6.38	(0.94, 17.84)	7.13	6.66	(-1.32, 16.27)

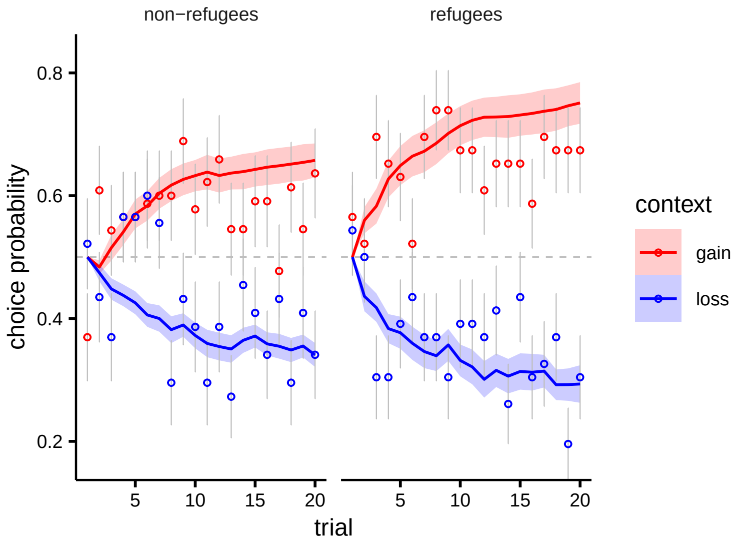

Figure 1: Plot of predicted and observed choices as a function of trial number.

1.2 Alternative model (value learning with altered reward-sensitivity)

Following the results of subsequent experiments, we tested a version of this model that is constrained such that refugees and non-refugee differ only in terms of a reward sensitivity parameter.

In brief, the model is essentially the same as the Q-learning model, with learned Q values updated according to

$$Q_{t+1}(c) = Q_t(c) + \eta \cdot \delta_t$$

where η is the learning rate. The updating is done separately for positive and negative feedbacks with possibly different learning rates η^+ and η^-.

The model differs from the standard one in the computation of the prediction error δ_t, specifically:

$$\delta_t = \begin{cases} r_t - Q_t(c) & \text{for } r_t \leq 0 \\ \lambda r_t - Q_t(c) & \text{for } r_t > 0 \end{cases}$$

Where λ is the reward sensitivity parameter, a multiplicative factor that scales the true value of *positive* rewards (that is, the value of coin's loss is not influenced by this parameter).

Note that due to the structure of the task (where positive rewards are only possible in the gain context), the effect of this parameter trade off, at least in part, with the η^+ parameter, the learning rate for the gain context. Because of this, we simplified the model by fixing the reward sensitivity parameter to 1 for the non-refugee children, and letting it as a free parameter for the refugee children. This approach is motivated by the specific aim of this analysis, which is to examine whether a difference in reward sensitivity alone can explain the pattern seen in the data. In other words, for fitting this model we fixed $\lambda_{\text{non-refugees}} = 1$ and estimated $\lambda_{\text{refugees}}$ as a free parameter.

The estimated parameters are shown in Table 4. The table suggests that differences in behaviour between groups could be explained assuming that refugee children perceived coins in the game to be about two and half times as valuable than non-refugee children.

Table 2: Group-level parameter of the alternative model. Estimates are the means of posterior distributions and intervals are Bayesian HPDI credible interval.

parameter	estimate	SE	95% CI
η^+	0.02	0.01	(0, 0.03)
η^-	0.03	0.03	(0, 0.06)
β^+	3.94	1.33	(1.92, 6.09)
β^-	3.06	1.07	(1.32, 4.65)
$\lambda_{\text{refugees}}$	2.68	1.21	(1.15, 4.19)

1.2.1 Alternative model's fit to data

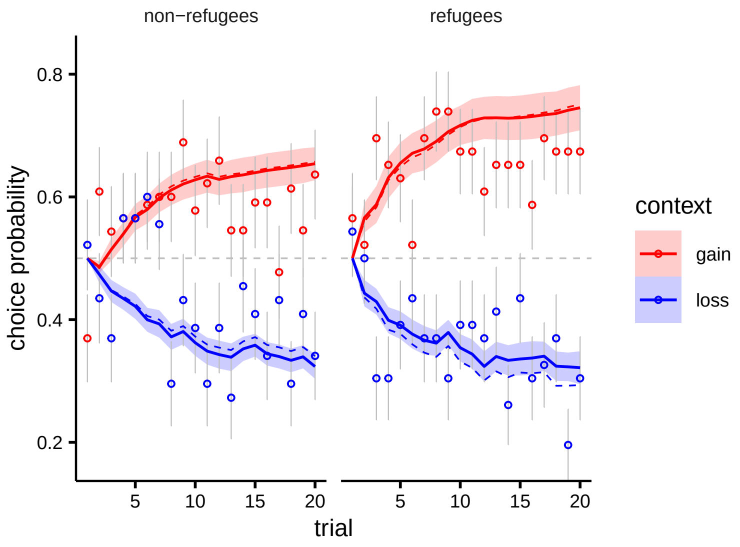

Figure 2: Plot of predicted and observed choices as a function of trial number. In this plot, continuous lines and bands are the prediction of the alternative model, while the dashed lines represents the predictions of the more complex Q-learning model.

1.3 Model comparison

To determine if the alternative model accounted for children's behavior in the task as well as, or better than, the standard value learning model, we compared the two models using the WAIC (Widely Applicable Information Criterion)[6]. This is an information criterion that can be interpreted in the same way as the AIC or BIC criteria, where lower values indicate a better model. The estimated WAIC for the standard Q learning model was 5934.8, whereas the value for the alternative model with only difference in reward sensitivity was 5926.4. This suggests that the alternative model provides a slightly better and more parsimonious account of the data.

1.4 Questionnaires

Table 3: Group means and differences for questionnaire scores and demographic variables. Group differences have been tested using Wilcoxon rank-sum test (also known as Mann–Whitney U test). The last column reports Bonferroni-corrected p-values.

Variable	U	p-value	mean non-refugees	mean refugees	adjusted p-value
**TEC**	184.5	0.000	0.429	6.370	0
**CRIES-8**	480.0	0.000	3.049	11.739	0
**AYMHS**	830.5	0.420	26.929	27.091	1
**Insecurity (HIDS)**	806.0	0.503	23.838	22.900	1
**Distress (HIDS)**	742.0	0.468	17.205	17.667	1
**Optimism (YLOT)**	1132.5	0.140	10.810	10.304	1
**PDCS (mother)**	161.0	0.788	9.765	9.750	1
**PDCS (father)**	84.0	0.145	9.733	10.188	1

1.5 Correlations of questionnaire scores with model parameters

The scatterplot matrix in Figure 3 illustrates the relationship between model parameters (of the standard value-learning model) and the main demographic variables and questionnaire scores

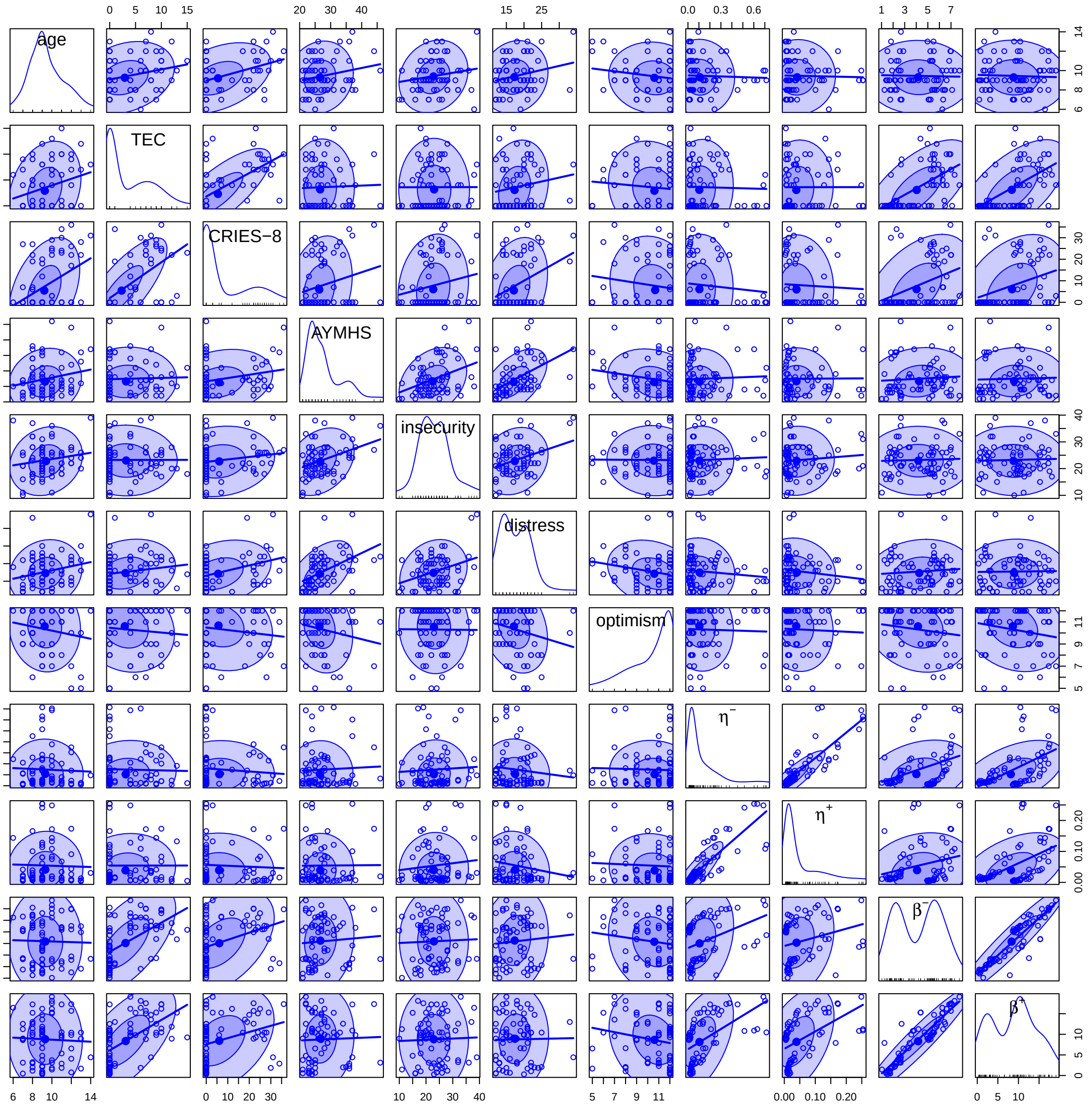

Figure 3: Scatterplot matrix showing correlations with model parameters.

2 Study 2: Explore/exploit decisions in a virtual foraging task

In addition to calculating the mean exit thresholds[7], as described in the main text, we analysed trial-by-trial behaviour in this task using a logistic regression model. In this model the probability of choosing to harvest the current tree was a logistic function of the expected number of apples:

$$P(\text{harvest})_t = \frac{1}{1 + e^{-(\beta_0 + \beta_1 \, [k \times \text{apples}_{t-1}])}}$$

where k is the average depletion rate (that is 0.88) and apples_{t-1} is the number of apples that the child obtained from the previous harvesting choice made in the current tree. For the first decision of each tree the quantity was set to 10. Note that this analysis assumes that children had, on average, a good estimate of the depletion rate. The model was fit at the group level using a hierarchical Bayesian approach. The model coefficient could differ across the two groups of children, and the parameters coding for the difference were given Gaussian priors centered at zero with standard deviations set to 1.

We also tested a variant of this model that included a lapse parameter controlling the probability of 'random' responses (i.e. independent of the apples received). In this model the probability was thus given by

$$P(\text{harvest})_t = \frac{\lambda}{2} + (1 - \lambda)\frac{1}{1 + e^{-(\beta_0 + \beta_1 \, [k \times \text{apples}_{t-1}])}}$$

Note that although the best strategy to approach this task is to keep track of the rewards (number of apples) obtained with each harvest (due to the between-trees variability in the amount of apples provided) a simpler alternative strategy could be to switch to a new tree after a fixed number of harvests - essentially ignoring the rewards. To verify that children paid attention to the rewards and used them to guide their choices, we fit an alternative model that implemented this alternative strategy. The model was similar to the logistic regression model described above with the difference that choice probability was taken to be a logistic function of the preceding number of harvests collected in the same tree rather than the expected number of apples at the next choice, that is:

$$P(\text{harvest})_t = \frac{1}{1 + e^{-(\beta_0 + \beta_1 \, [\text{n. previous harvests}])}}$$

2.1 Model estimation & comparison

We fitted the models by using Hamiltonian Monte Carlo (HMC) sampling as implemented in Stan and its R interface[4] to estimate the posterior distribution of the parameters. We ran 4 chains of 4000 samples each (2000 burn-in); convergence was assessed by verifying that there were no divergent transitions and that $\hat{R} \approx 1$ for all parameters[5]. We compared the models' fit to the data using the WAIC criterion[6]. The results are shown in Figure 4. The fit of the best model to the data is shown in Figure 5.

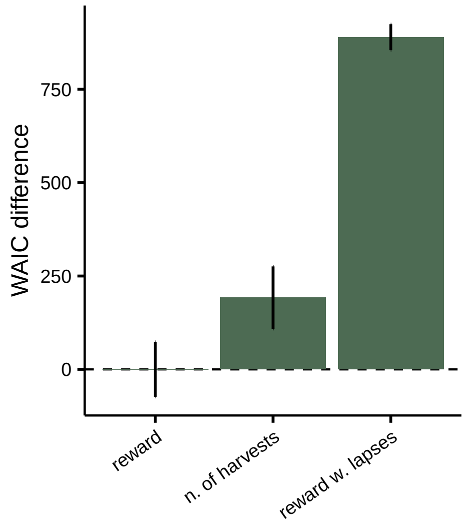

Figure 4: Model comparison. The bars show the WAIC of each model as a difference from the best model (note: smaller WAIC indicate a better model). Error bars are standard errors.

Table 4: Group-level parameter of the best-fitting model, that is the model in which harvesting choices were predicted solely from the expected reward (number of apples). Estimates are the means of posterior distribution and intervals are Bayesian HPDI credible interval.

	Non-refugees			Refugees		
parameter	estimate	SE	95% CI	estimate	SE	95% CI
β_0	-4.49	0.57	(-5.39, -3.58)	-4.58	0.68	(-5.69, -3.52)
β_1	0.84	0.07	(0.73, 0.94)	0.85	0.08	(0.72, 0.97)

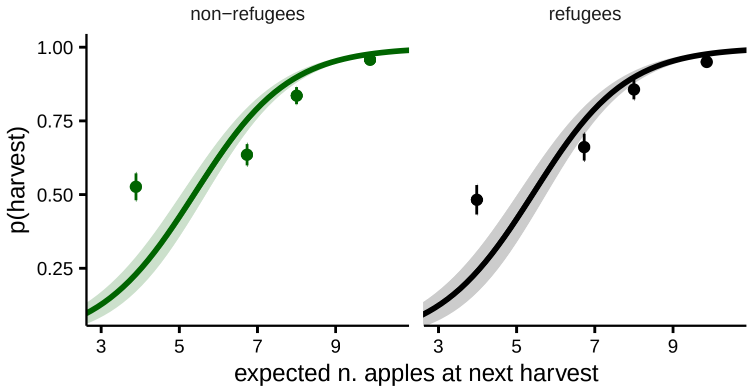

Figure 5: Group-level choice functions estimated from the single trial analysis, plotting the probability of harvesting the current tree as a function of the expected number of apples at the next harvest. Dots represent the binned data. Error bars represent standard errors across children.

2.2 Questionnaires

Table 5: Group means and differences for questionnaire scores and demographic variables. Group differences were tested using Wilcoxon rank-sum test (also known as Mann–Whitney U test). The last column reports Bonferroni-corrected p-values.

Variable	U	p-value	mean non-refugees	mean refugees	adjusted p-value
**TEC**	16.5	0.000	1.316	6.571	0.000
**CRIES-8**	263.5	0.760	5.083	4.905	1.000
**AYMHS**	1216.5	0.034	25.522	27.630	0.237
**Insecurity (HIDS)**	1367.0	0.247	20.151	21.349	1.000
**Distress (HIDS)**	1574.0	0.108	17.038	19.167	0.756
**PDCS (mother)**	2196.5	0.182	9.734	9.408	1.000
**PDCS (father)**	1862.0	0.617	9.403	9.370	1.000

2.3 Correlations of questionnaire scores with model parameters

The scatterplot matrix in Figure 6 illustrates the relationship between model parameters (of the best model, β_0 and β_1), age, questionnaire scores and other summary statistics of behaviour in the task.

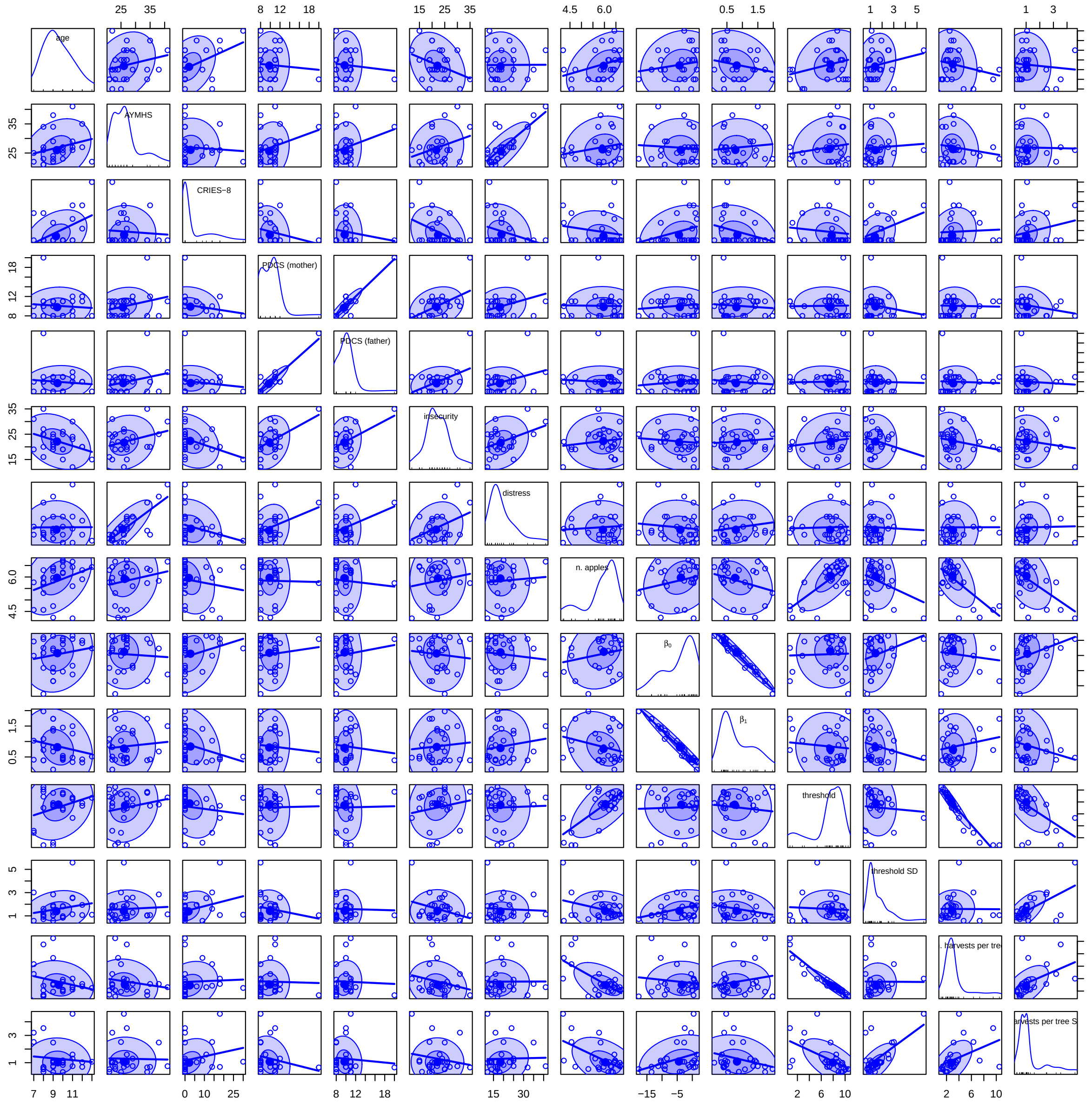

Figure 6: Scatterplot matrix showing correlations with model parameters.

3 Study 3: Comparing certain and uncertain rewards

Study 3 introduced a novel experimental protocol where children chose between a 'sure' (S) door that always gave 1 coin and two 'risky' (R) doors with varying probabilities of yielding a larger reward of 2 coins. This protocol required children to choose between a smaller, certain reward and a larger, uncertain reward, thereby probing not only children's learning under uncertainty but also their risk preferences.

Initially, children were expected to choose the S door as frequently as the R doors, since without knowing the probabilities of the risky doors, the subjective expected value of picking any of the risky doors is the same as that of choosing the sure door (that is, 1 coin). However, as children gathered information about the higher reward probability of one R door, their choices were predicted to shift towards it. We paid particular attention to how outcomes influenced the subsequent probability of choosing the S door in the modeling of this task. Each child completed 10 practice trials and 30 experimental trials (see main text for details on the procedure).

Due to the novelty of the protocol, we considered several alternative computational models:

- *Naive value learning*: This model is formally identical to that used in the analysis of Experiment 1 (reinforcement learning with a softmax choice function), except it did not include separate parameters for loss and gain contexts (since this experiment did not include a loss context). The model is naive because it ignores that the value of the S door is known in advance and learns it the same way as the value of the other doors. This can be seen as a baseline model, suitable for a child that did not understand the task instructions.

- *Value learning*: This model is identical to the naive value learning model, except that the value of the S door is set to a fixed value, estimated as a free parameter.

- *Dual value learning*: This model implements two parallel learning processes. The first is analogous to the value learning model and is directed toward learning which of the two R doors has a higher expected value. The second decision process focuses on learning whether the expected value of choosing one of the R doors is higher or lower compared to the certain reward provided by the S door. This model implements a plausible strategy for an agent that knows the structure of the task (i.e., that the value of the S door is fixed) but does not know the probabilities associated with the R doors and therefore seeks to determine whether they have a larger or smaller expected value than the S door.

- *Value learning with reward-dependent stickyness*: This model modifies the value learning model by adding stickiness parameters that account for the tendency to repeat choices and an additional reward-dependent stickiness parameter. This can be understood as implementing a simpler, memoryless version of the second learning process in the dual value learning model. The decision between the two R doors is governed by a reinforcement learning model, identical to the previous model. However, the propensity to choose between the S and R doors is regulated by a memoryless process that depends only on the reward observed in the previous trial. In other words, this could be seen as an implementation of a win-stay-lose-shift strategy, where a parameter quantifies the sensitivity to winning a risky reward, determining how much the probability of choosing the R door again increases after winning 2 coins compared to not winning any coins.

The following sections provide the mathematical details of each model and illustrate their fit to the data. In all cases, the models were fit at a group level using a hierarchical Bayesian approach. Each model was fit in Stan, using 4 chains of 4000 samples each; convergence was assessed by verifying that there were no divergent transitions and that $\hat{R} \approx 1$ for all parameters[5].

3.1 *Naive* value learning model

This model assumes that on each trial t the subjects make a choice $c_t \in \{1, 2, 3\}$ (the three doors) and observe a "reward" $r_t \in \{0, 2, 1\}$. The subject maintains and updates estimates of the value (the estimated expected reward) of each choice option (the so-called Q-values). The Q-values are updated according to

$$Q_{t+1}(c) = Q_t(c) + \eta\, \delta_t$$

where η is the learning rate. δ_t is the reward prediction error, calculated as

$$\delta_t = r_t - Q_t(c)$$

A softmax function is used to transform values to choice probabilities

$$p(c_t = i) = \frac{e^{\beta Q_t(c=i)}}{\sum_{j=1}^{3} e^{\beta Q_t(c=j)}}$$

where β is an "inverse temperature" parameter that controls the randomness of the choices.

Note that the equations above would describe the model for a single child; in the analysis the parameters of all children were estimated jointly using a hierarchical Bayesian approach, corresponding to assuming that individual parameters have the following multivariate Gaussian distribution

$$\begin{bmatrix} \Phi^{-1}(\eta_i) \\ \log(\beta_i) \end{bmatrix} \sim \mathcal{N}(\mu, \Sigma)$$

Additionally we had parameter coding for group-level differences for both learning rate and choice variability. The parameters were defined on the transformed, unbounded values of the parameters, and were given as prior Gaussian distributions with mean of zero and standard deviations of 1.

The model captures average choice rates reasonably well for the different alternatives. The model captures reasonably well the choice probability of Jordanian children, but less so those of Syrian children, as shown in Figure 7. However, it does not capture at all the sequential effects as shown in Figure 8: the plot shows that whereas children in both groups were alternating between risky and sure doors across consecutive trials (as shown for example by the fact that after a S door choice, corresponding to a sure 1 coin win, participants were less likely than chance to select the S door again, whereas the model predict the opposite). The estimated group-level parameter values are reported in Table 6.

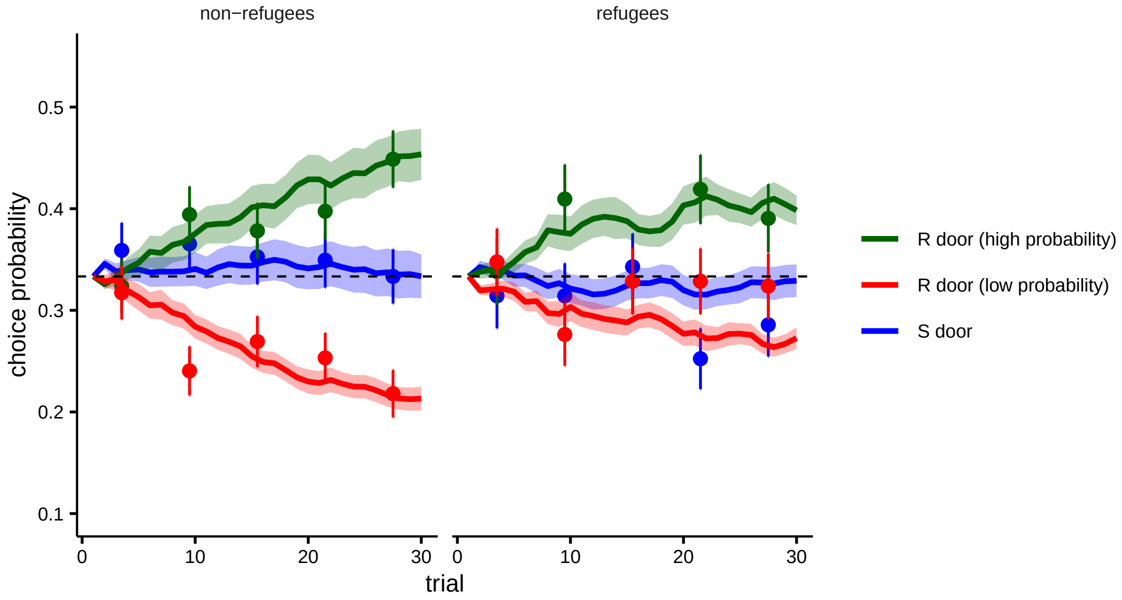

Figure 7: Plot of predicted and observed choices as a function of trial number. In this plot, continuous lines and bands are the predictions of the naive value learning model. Dots represents the average of choices in 6 trials.

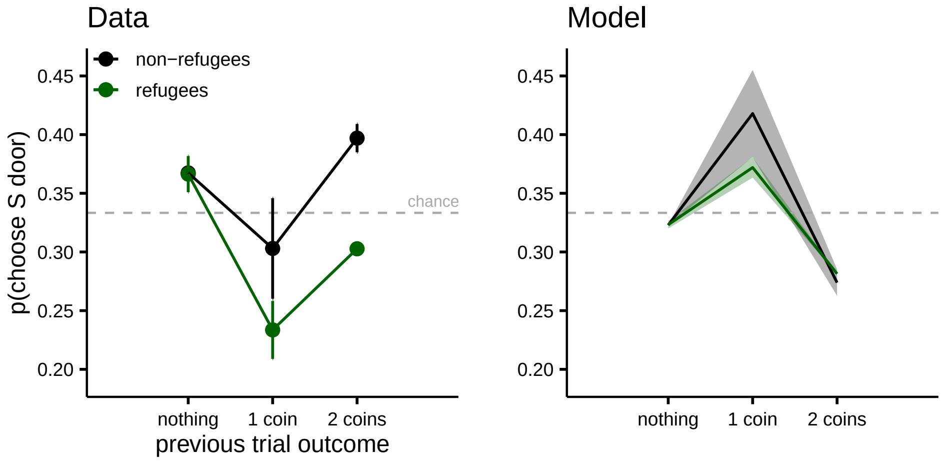

Figure 8: Plot of predicted and observed fraction of S door choices as a function of the outcome of previous choice.

Table 6: Group-level parameter. Estimates are the means of posterior distribution and intervals are Bayesian HPDI credible interval.

	Non-refugees			Refugees			Group differences		
parameter	estimate	SE	95% CI	estimate	SE	95% CI	difference	diff. SE	diff. 95% CI
η	0.10	0.05	(0.03, 0.16)	0.19	0.08	(0.07, 0.3)	0.08	0.08	(-0.05, 0.21)
β	1.54	0.48	(0.86, 2.24)	0.89	0.28	(0.48, 1.26)	-0.66	0.48	(-1.37, 0.05)

3.2 Value-learning (with fixed value for S door)

This model works just like the previous 'naive' value learning model (3.1), with the exception that the (Q) value of the S door is set to a fixed value that is not updated during learning. This modelling assumption reflects the fact that children were told that this door always returns 1 coin. This model thus has an additional free parameter per participant: the Q-value for the S door. While this model somewhat still captures the average choices over time, as shown in Figure 9, it fails to capture sequential effects, see Figure 10. The estimated group-level parameter values are reported in Table 7.

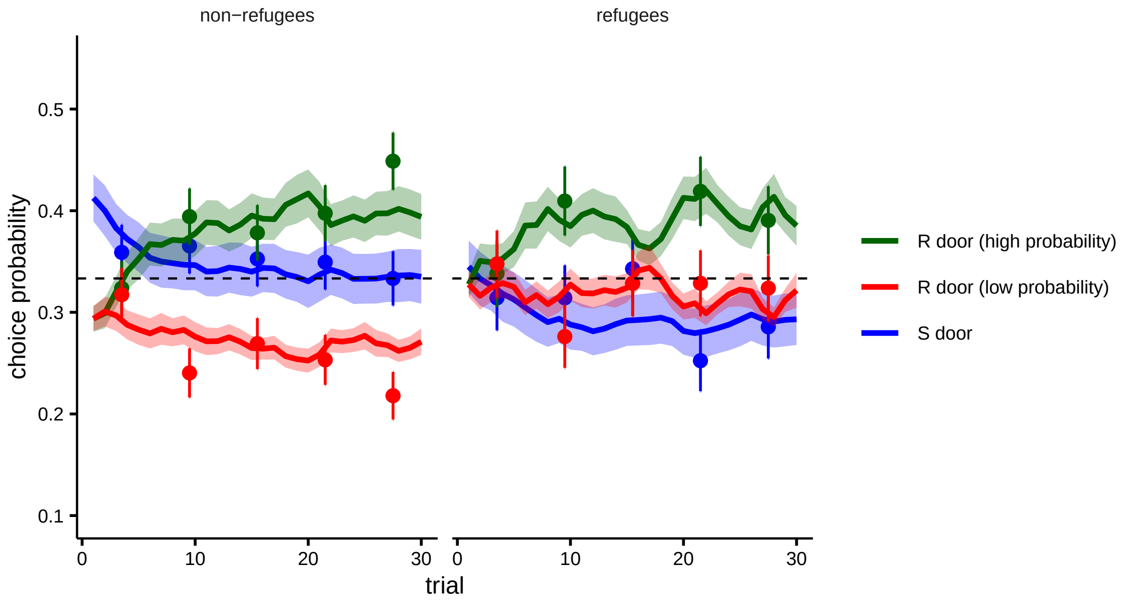

Figure 9: Plot of predicted and observed choices as a function of trial number. In this plot, continuous lines and bands are the prediction of the naive value learning model.

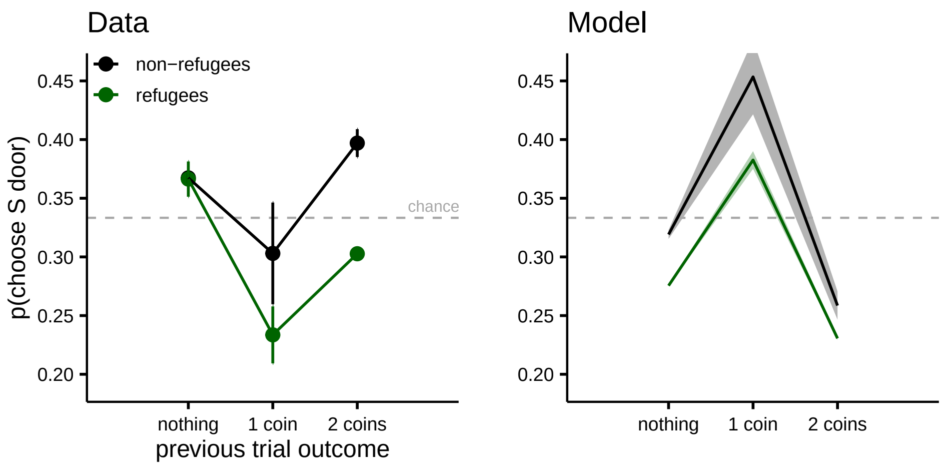

Figure 10: Plot of predicted and observed fraction of S door choices as a function of the outcome of previous choice.

Table 7: Group-level parameters. Estimates are the means of posterior distribution and intervals are Bayesian HPDI credible interval.

	Non-refugees			Refugees			Group differences		
parameter	estimate	SE	95% CI	estimate	SE	95% CI	difference	diff. SE	diff. 95% CI
η	0.47	0.15	(0.23, 0.72)	0.55	0.20	(0.23, 0.87)	0.07	0.19	(-0.23, 0.39)
β	0.48	0.10	(0.33, 0.64)	0.39	0.10	(0.23, 0.53)	-0.09	0.09	(-0.23, 0.05)
$Q(S)$	0.78	0.32	(0.28, 1.29)	0.33	0.41	(-0.27, 1.02)	-0.45	0.45	(-1.14, 0.31)

3.3 Dual value learning model

This model is a extension of the simpler model considered so far that aims to more directly capture the sequential effects we have observed. In this model 2 independent learning processes occur simultaneously: one about learning which of the 2 'risky' (R) doors have higher expected reward, and another that learns the relative value of going for any of the two R doors vs the S door. Additionally, the model has a stickyness parameter that codes for the tendency of repeating/alternating decisions of attempting one the R doors vs going for the S door.

Formally, in the model there is first a learning process that determines the values of the risky doors and choice probabilities for the 2 risky options, conditioned on not choosing the sure one.

The 2 Q-values of the R doors are updated after each R choice according to

$$Q_{t+1}\left(c \in R\right) = Q_t\left(c \in R\right) + \eta\, \delta_t$$

where η is the learning rate. δ_t is the reward prediction error, calculated as

$$\delta_t = r_t - Q_t\left(c \in R\right)$$

A softmax function is used to transformate values to choice probabilities

$$p\left(c_t = i \mid R\right) = \frac{e^{\beta Q_t(c=i,\, c\in R)}}{\sum_{j=1}^{2} e^{\beta Q_t(c=j,\, c\in R)}}$$

The Q-value of the S door is set to a fixed value, $Q(S)$, which is not updated.

The value of attempting a 'risky' choice, $Q(R)$, is instead updated after each risky choice. Essentially this would result in choosing more frequently the risky doors as the learning progresses and information about which of the two doors is better is gathered. Formally

$$\begin{aligned} Q_{t+1}\left(R\right) &= Q_t\left(R\right) + \eta_R \cdot \delta_t \\ \delta_t &= r_t - Q_t\left(c \in R\right) \end{aligned}$$

Additionally, the model has a 'stickyness' parameter ω that represents the tendency of repeating or alternating choices of R and S doors. This is obtained by modifying the Q values according to the previous choice. Formally, the modified values (notated with Q') are calculated as

$$Q'_t(S) = \begin{cases} Q_t(S) + \omega & \text{if } c_{t-1} \equiv S \\ Q_t(S) & \text{if } c_{t-1} \not\equiv S \end{cases}$$

for the S door and

$$Q'_t(R) = \begin{cases} Q_t(R) + \omega & \text{if } c_{t-1} \in R \\ Q_t(R) & \text{if } c_{t-1} \notin R \end{cases}$$

The probability of 'risky' (R) is given again by a softmax function

$$p_t\left(R\right) = \frac{1}{1 + e^{-\beta_R \left[Q'_t(R) - Q'(S)\right]}}$$

Finally, applying the law of total probability one obtains the unconditional probabilities of choosing either of the R doors

$$p_t\left(c_t = i\right) = p_t\left(c_t = i \mid R\right) \times p_t\left(R\right)$$

for the two R doors, and

$$p_t\left(S\right) = 1 - p_t\left(R\right)$$

For the S door.

This model has greater flexibility and a larger number of free parameters (6 per participant). However, while it does a better job in capturing sequential effects - see Figure 14 - this model does not account well for group differences, whereby the two groups respond similarly after having chosen an R door and having received no reward, but differently after having received two coins (the refugees are more likely than chance to choose again an R door, whereas the non-refugees are more likely to switch to the S door regardless of the outcome). Furthermore, one critical consequence of this model is that the fraction of predicted S choices decreases during the task, however there is little evidence for such a trend in the data. We reasoned that a simpler model, without a second learning process, but with a stickiness parameter that is modulated by the reward in the previous trial might do a better job in explaining the data. The estimated group-level parameters are shown in Table 8.

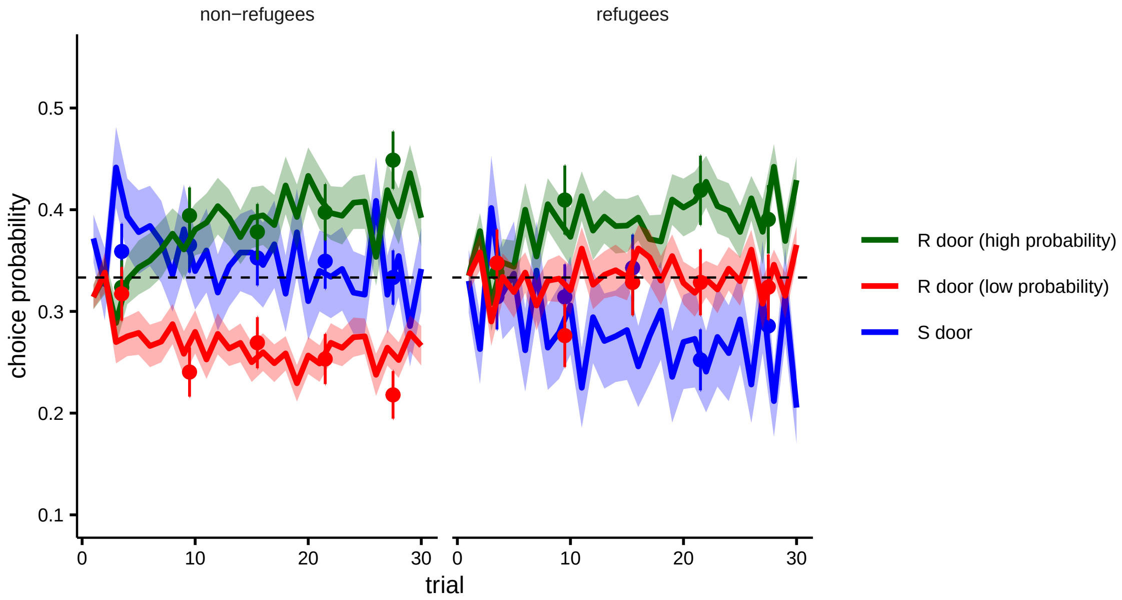

Figure 11: Plot of predicted and observed choices as a function of trial number. In this plot, continuous lines and bands are the prediction of the dual value learning model.

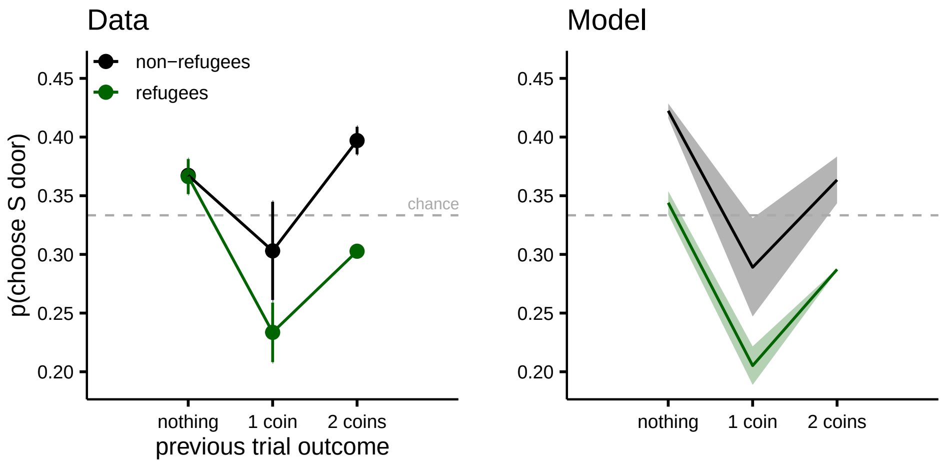

Figure 12: Plot of predicted and observed fraction of S door choices as a function of the outcome of previous choice.

Table 8: Group-level parameter. Estimates are the means of posterior distribution and intervals are Bayesian HPDI credible interval.

	Non-refugees			Refugees			Group differences		
parameter	estimate	SE	95% CI	estimate	SE	95% CI	difference	diff. SE	diff. 95% CI
η	0.42	0.16	(0.15, 0.66)	0.54	0.25	(0.17, 0.96)	0.12	0.23	(-0.23, 0.5)
β	0.60	0.17	(0.31, 0.85)	0.34	0.15	(0.12, 0.55)	-0.26	0.16	(-0.52, -0.01)
η_R	0.10	0.07	(0.01, 0.19)	0.25	0.12	(0.05, 0.43)	0.15	0.12	(-0.02, 0.34)
$Q(S)$	-0.56	0.24	(-0.91, -0.19)	-0.71	0.31	(-1.16, -0.24)	-0.15	0.29	(-0.6, 0.29)
β_R	1.07	0.25	(0.66, 1.44)	1.12	0.27	(0.69, 1.54)	0.05	0.19	(-0.25, 0.34)
ω	-0.79	0.24	(-1.14, -0.43)	-0.86	0.27	(-1.26, -0.43)	-0.07	0.25	(-0.48, 0.31)

3.4 Value learning with reward-dependent stickiness

This final model can be seen as a simpler, memoryless version of the previous model. Here, the probability of repeating an R choice is modulated by the reward observed in the previous choice (akin to a win-stay-lose-shift strategy). The model works as follows: as in previous models, at each trial t the subjects make a choice $c_t \in \{1, 2, 3\}$ (the three doors) and observe a "reward" $r_t \in \{0, 2, 1\}$. The subject maintains and updates estimates of the value (the estimated expected reward) of the risky choice options (the so-called Q-values). The Q-values of the two risky doors, notated with $Q(c_i \in R)$, are updated according to

$$Q_{t+1}\left(c \in R\right) = Q_t\left(c \in R\right) + \eta\, \delta_t$$

where η is the learning rate. δ_t is the reward prediction error, calculated as

$$\delta_t = r_t - Q_t\left(c \in R\right)$$

There is no uncertainty about the value of the 'sure' door (it is known from the beginning that it gives always 1 coins), therefore its Q-value ($Q(c \equiv S)$) is set to a fixed (unknown) value that is estimated for each kid.

Differently from the simplest learning model, here the Q-values are adjusted in each trial based on the outcome of previous trials. This adjustments uses 2 additional parameters. A stickiness parameter ω code for the tendency of repeating (or not) a previous decision to pick the sure or a risky alternative. An additional parameter ρ further modifies the tendency to repeat 'risky' choices after a risky choice, based on the previous outcome.

$$Q'_t(c \in R) = \begin{cases} Q_t(c \in R) + \omega + \rho & \text{if } c_{t-1} \in R \text{ and } r_{t-1} = 2 \\ Q_t(c \in R) + \omega - \rho & \text{if } c_{t-1} \in R \text{ and } r_{t-1} = 0 \\ Q_t(c \in R) & \text{if } c_{t-1} \notin R \end{cases}$$

Similarly, the value of the 'sure' door is adjusted depending on whether participants previously choose 'sure' or not.

$$Q'_t(S) = \begin{cases} Q_t(S) + \omega & \text{if } c_{t-1} \equiv S \\ Q_t(S) & \text{if } c_{t-1} \not\equiv S \end{cases}$$

A softmax function is used to transformate values to choice probabilities

$$p\left(c_t = i\right) = \frac{e^{\beta Q'_t(c=i)}}{\sum_{j=1}^{3} e^{\beta Q'_t(c=j)}}$$

where β is an "inverse temperature" parameter that control the randomness of the choices.

This model thus

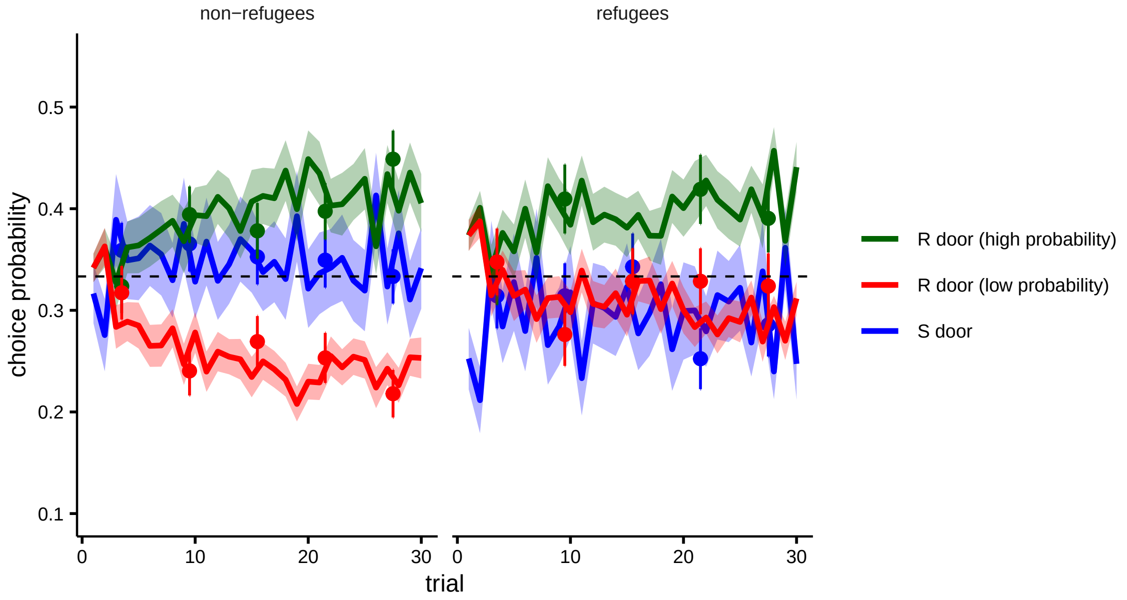

Figure 13: Plot of predicted and observed choices as a function of trial number. In this plot, continuous lines and bands are the prediction of the value learning model with reward-modulated stickiness.

This model perfectly predicts the sequential effects, as shown in figure 14, as well as the difference between refugees and non-refugees. In particular, the model predictions replicates the pattern that after choosing an R door and winning two coins, refugees choose an R door again more frequently than chance (as indicated by probability of choosing the S door below the chance level of 1/3), whereas non-refugees are instead more frequently choosing the S door. The estimated group-level parameters for this model are shown in Table 9.

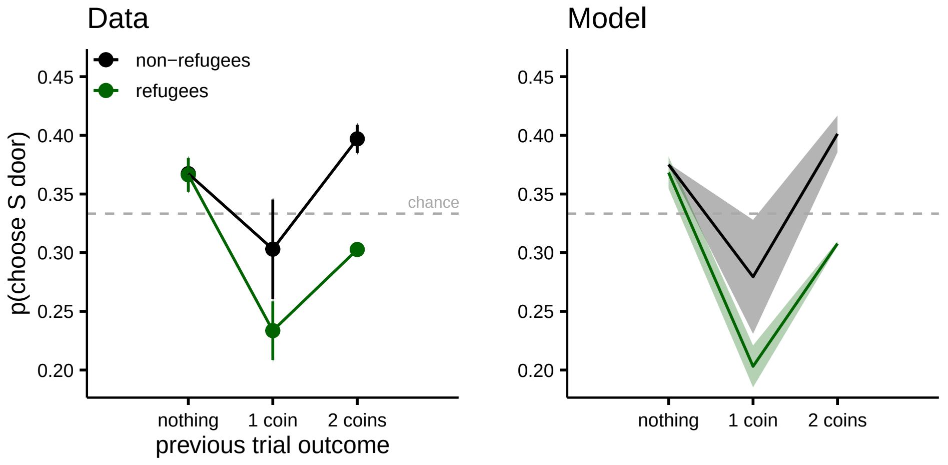

Figure 14: Plot of predicted and observed fraction of S door choices as a function of the outcome of previous choice (the model is is the value learning model with reward-modulated stickiness).

Table 9: Group-level parameter. Estimates are the means of posterior distribution and intervals are Bayesian HPDI credible interval.

	Non-refugees			Refugees			Group differences		
parameter	estimate	SE	95% CI	estimate	SE	95% CI	difference	diff. SE	diff. 95% CI
η	0.45	0.12	(0.25, 0.63)	0.35	0.14	(0.15, 0.57)	-0.09	0.14	(-0.33, 0.12)
β	0.80	0.15	(0.57, 1.03)	0.68	0.14	(0.45, 0.89)	-0.13	0.12	(-0.31, 0.07)
$Q(S)$	-0.45	0.27	(-0.88, -0.05)	-0.91	0.36	(-1.47, -0.37)	-0.46	0.38	(-1.06, 0.13)
ρ	-0.46	0.20	(-0.77, -0.14)	0.18	0.24	(-0.19, 0.58)	0.65	0.30	(0.13, 1.09)
ω	-0.96	0.27	(-1.38, -0.54)	-0.98	0.34	(-1.48, -0.42)	-0.02	0.35	(-0.55, 0.54)

3.5 Model comparison

We compared the fits of the four models presented in the previous section using the WAIC criterion[6]. The results, shown in Figure 15, indicate that the *value learning with reward-dependent stickiness* model provides the best account of the data.

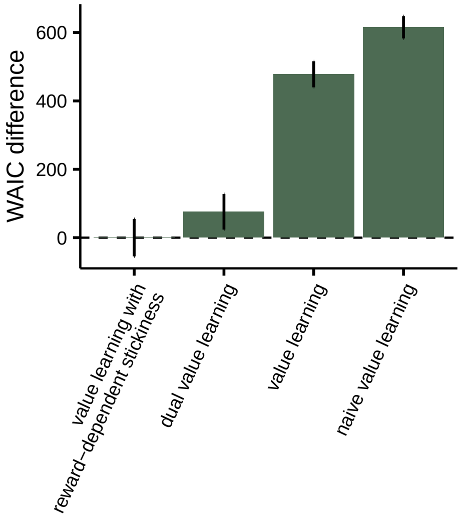

Figure 15: Model comparison. The bars show the WAIC of each model as difference from the best model (note: smaller WAIC indicate a better model). Error bars are standard errors.

3.6 Questionnaires

Table 10: Group means and difference for questionnaire scores and demographic variables. Group difference have been tested using Wilcoxon rank-sum test (also known as Mann–Whitney U test). The last column reports Bonferroni-corrected p-values.

Variable	U	p-value	mean non-refugees	mean refugees	adjusted p-value
**TEC**	148.0	0.000	1.571	6.000	0.000
**CRIES-8**	96.5	0.920	6.000	6.545	1.000
**AYMHS**	400.0	0.054	24.878	26.296	0.377
**Insecurity (HIDS)**	480.0	0.642	19.465	20.292	1.000
**Distress (HIDS)**	577.5	0.378	16.617	17.607	1.000
**PDCS (mother)**	789.0	0.197	10.062	9.643	1.000
**PDCS (father)**	597.0	0.885	9.533	9.538	1.000

3.7 Correlations of questionnaire scores with model parameters

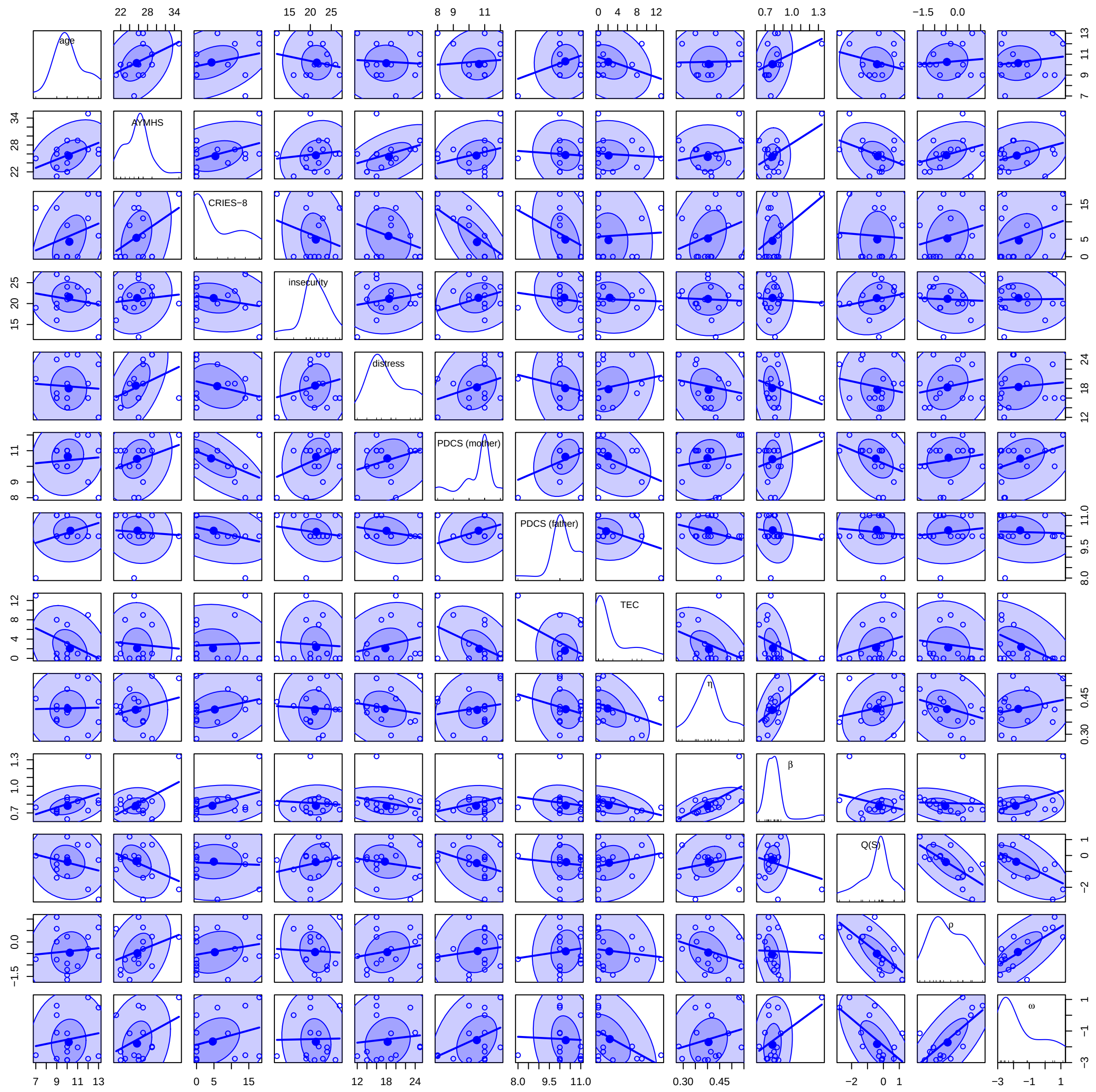

Figure 16: Scatterplot matrix showing correlations of questionnaires and model parameters.

4 Study 4: Reward sensitivity using a perceptual decision-making task

4.1 Methods

Participants. Participants were Syrian refugee (n = 54, 26 girls) and Jordanian non-refugee children (n = 58, 25 girls) aged between 7 and 13 years old (M age = 10.55, SD = 1.64) recruited through the collaborating NGO Taghyeer. participants recruitment for this experiment proved more difficult than anticipated, and we were able to collect only about half of the intended sample size as indicated in the pre-registration (available at osf.io/y8dgw). Children took part in the study in August 2022 in the East Amman Charitable Community Centre in the Southern Marka region of Amman. The study was approved by the Queen Mary University Research Ethics Boards (QMERC22.115). Caregivers gave their informed written consent and children their verbal assent prior to taking part in the study. Participating families were reimbursed 5JD for travel expenses.

Questionnaires. We used validated questionnaires either developed in or adapted to Arabic administered with Kobo (Kobo toolbox, 2021) to assess children's wellbeing and mental health. The experimental task was run in Matlab (Mathworks) on a computer laptop. Children reported on their symptoms of PTSD (CRIES-8[8]), internalising problems (AYMHS[9]), and feelings of insecurity (HIDS[10]). Children's primary caregivers reported on their children's exposure to war-related traumatic events (TEC[11]) as well as children's internalising, externalising, and attention problems (PSC-17[12]). The questionnaires and the computer task instructions were provided in Arabic by female fieldworkers who explained the task carefully and verified that the children understood the instructions. The fieldworkers administered the questionnaires by reading all measures aloud to the children and using visual scales where appropriate.

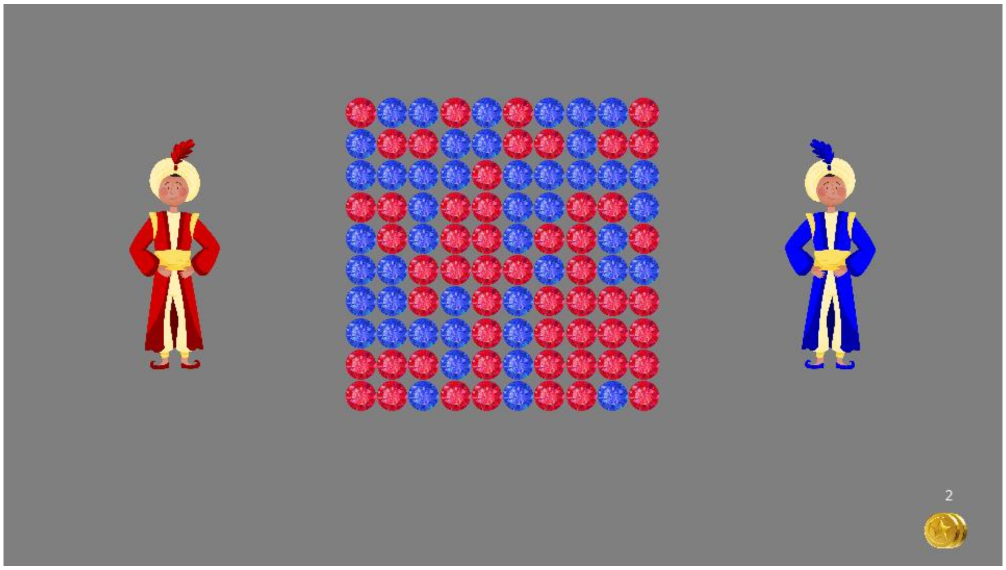

Figure 17: Task screenshot

Procedure. Children took part in this perceptual task, that was presented as a game to determine the prevalence of either red or blue "gems" within a 10 x 10 array, see figure 17. The primary challenge was the closely matched ratio of 54:46 between the two gem colors. To ensure that the

children were actively engaged, we interspersed catch trials within the primary trials that had an easily discernible ratio of 85:15 between the coloured gems. Each child was expected to complete 60 trials. However, if a child failed to register a response within a strict 2-second window, the specific trial was repeated – with the caveat that no child could exceed a total of 120 trials. Prior to the experimental trials, children underwent a practice round of 15 trials to familiarise themselves with the task. Notably, during these practice rounds, the reward probability for a correct answer was evenly distributed between the two possible outcomes, unlike in the main task where a correct answer was rewarded 80% of the time for one gem color ("rich" stimulus) and 20% for the other ("lean" stimulus). Children who found the practice trials unclear were given the opportunity to repeat them. The aim of this experiment was to specifically examine reward sensitivity between Syrian refugees children and age-matched Jordanian controls. This methodology and reward system was adapted from the original design by Pizzagalli et al[13], and more specifically on a variant that has been used recently to assess reward sensitivity in children with ADHD[14].

The fieldworkers were instructed to present the following background story to introduce children to the task: *"Prince Blue and prince Red are dividing a treasure between themselves. The treasure consists of many boxes of gems. They decide that prince Red will get the boxes that contains more red gems (rubies), and prince Blue with get the boxes that contains more blue gems (sapphires). There are an equal number of boxes with more red or blue gems. The princes are very busy, so are in a hurry, and your task is to help them by indicating very quickly whether a box contains more red or blue gems. You will have 2 seconds to make your decision. If you choose correctly, the prince may reward you with a coin – although sometimes they forget to give the coin."*

4.2 Analysis

Model-agnostic analysis. A response bias was calculated using the formula from Pizzagalli et al.[13], that is

$$\text{bias} = \frac{1}{2}\log\left(\frac{n(c_r \mid s_r)\ n(c_r \mid s_l)}{n(c_l \mid s_r)\ n(c_l \mid s_l)}\right)$$

where s_r and s_l denote the presentation of the "rich" and "lean" stimuli (corresponding to the gem colour with high and low reward probability, respectively); c_r and c_l the participant's choices of rich and lean stimuli, respectively; and $n(c_r \mid s_r)$, for example, would be the number of trials in which the majority of gems were of the "rich" colour was presented (that is, the display contained more gems of the color associated with high reward probability) and participant responded correctly by chosing the 'rich' colour. Each count was augmented by 0.5 to avoid numerical instabilities.

Computational Modelling. Based on Huys et al[15], participants' choices are evaluated against two weights - the current stimulus and the learned value. For example, if the stimulus presented at trial t is s_t, the weight of choosing "rich" at the same trial is notated as $W_t(c_r, s_t)$. The mapping from weight to probability is done via a softmax sigmoid function:

$$p(c_r \mid s_t) = \frac{1}{1 + e^{-(W_t(c_r,s_t) - W_t(c_l,s_t))}}.$$

The weight itself is a function of both the stimulus presented, and the learned value:

$$W_t(c_t, s_t) = \gamma I(c_t, s_t) + Q_t(c_t, s_t)$$

where $\gamma I(c_t, s_t)$ correspond to the participant's ability to perform the discrimination task according to instructions; specifically $I(c_t, s_t) = 1$ if stimulus s_t require the choice c_t (i.e. if participants chose 'rich' when the rich stimulus is presented, or 'lean when the lean stimulus is presented.); and γ is a free parameter (larger values corresponds to more frequent correct responses).

Instead, $Q_t(c_t, s_t)$ is a learned reward value (Q value) that is updated across trials according to a standard Q-learning update rule:

$$Q_{t+1}(c_t, s_t) = Q_t(c_t, s_t) + \epsilon\ \underbrace{(\rho r_t - Q_t(c_t, s_t))}_{\text{reward prediction error}}$$

where r_t is the reward observed after making choice c_t at trial t. ϵ and ρ are free parameters, corresponding to the learning rate and the reward sensitivity, respectively. Note that the reward sensitivity parameter is essentially a factor that scales the true value of the reward: values of $\rho > 1$ would indicated a heightened sensitivity to reward during learning.

Following the pre-registration, the above model was fit using a Bayesian multilevel approach, with participant-specific random effects for each parameter and a fully parameterized variance-covariance matrix. The pre-registration indicated that we hypothesized a difference in the reward sensitivity parameter ρ, so we included also a group-level (fixed-effects) parameter coding for the difference in ρ between refugees and non-refugees.

4.3 Results

Demographics. There was no significant difference in the mean age between the two groups: non-refugees had a mean age of 10.62 (SD 1.62) years, whereas refugees had a mean age of 10.30 (SD 1.59) years; $t(110)=1.0424$, $p=0.2995$. The Bayes factor (null/alternative) for this comparison was 4.10 (Bayes factors for this and other simple comparisons were computed with a *JZS* prior[16]). The proportion of females between the groups also did not differ significantly: non-refugees had a proportion of 0.43, while refugees had a proportion of 0.48, $\chi^2(1)=0.12$, $p=0.73$; the Bayes factor (null/alternative) for this was 8.47.

Questionnaires. We used the Wilcoxon test to investigate potential differences between refugee and non-refugee children's trauma exposure and mental health outcomes. We found that the refugee group (M = 2.90) experienced a significantly higher number of traumatic events related to war and displacement (caregiver-reported) than non-refugees (M = 0.41), with the Bonferroni corrected p = .001. The groups did not differ on any mental health outcomes, both reported by the children themselves and by the caregiver - see table 11.

Table 11: Group means and difference for questionnaire scores and demographic variables. Group difference have been tested using Wilcoxon rank-sum test (also known as Mann–Whitney U test). The last column reports Bonferroni-corrected p-values.

Variable	U	p-value	mean non-refugees	mean refugees	adjusted p-value
**TEC**	411.5	0.000	0.405	2.902	0.001
**CRIES-8**	112.0	0.244	27.062	21.909	1.000
**AYMHS**	1328.5	0.891	30.333	30.140	1.000
**Insecurity (HIDS)**	1269.5	1.000	32.056	31.894	1.000
**PSC (internalising)**	561.0	0.035	3.611	4.907	0.247
**PSC (attention)**	618.0	0.337	5.061	5.767	1.000
**PSC (externalising)**	824.0	0.661	5.500	5.341	1.000

Model-agnostic analyses. Across all participants, the bias[13] was positively associated with the final score (total number of coins obtained during the task), Pearson's r=0.32, 95% CI [0.14, 0.48]; see figure 18. However, we did not find a significant difference between groups, $t(110)$=0.28, p=0.78. The Bayes factor (null/alternative) for this comparison was 6.59. Average bias values for each of the two groups are reported in table 12. The distribution of scores for the two groups is shown in figure 19. On average refugees obtained 23.8 coins (Std. 6.2), and non-refugees obtained 22.2 coins (5.1); the difference was not statistically significant $t(110)$=1.48, p=0.14.

Table 12: Average bias

			95% CI	
group	bias	SE	lower	upper
non-refugees	0.118	0.090	-0.035	0.296
refugees	0.158	0.105	-0.063	0.358

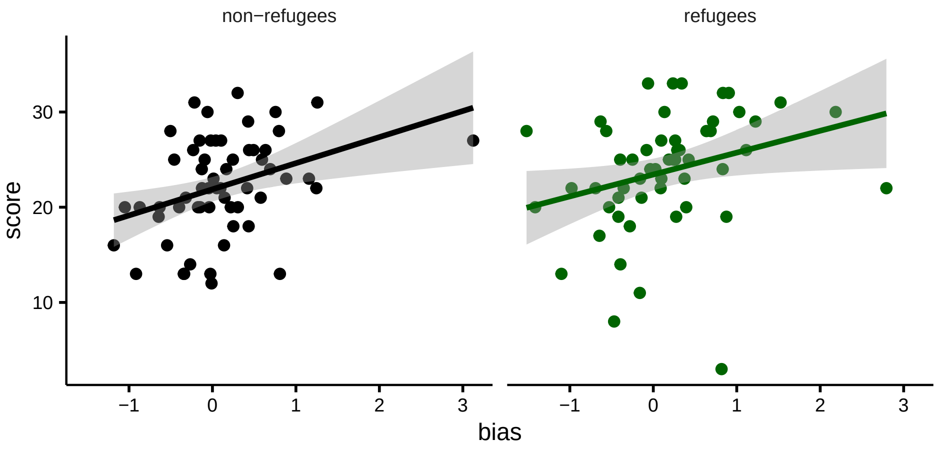

Figure 18: Relationship between response bias and maximum score achieved in the task

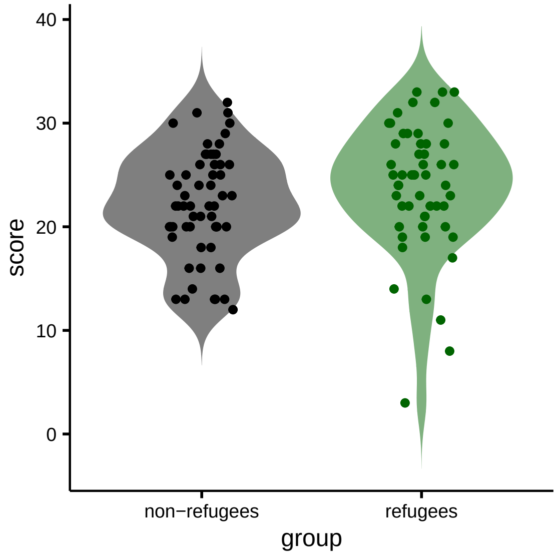

Figure 19: Distribution of scores (n. of coins), plotted separately for the two groups.

Computational modelling. The estimated group-level values for the computational models are shown in table 13. Although numerically the group-level reward sensitivity parameter ρ was larger in the refugee group, this difference is not statistically significant as indicated by the fact that the 95% Bayesian credible interval of this difference included zero. Note also that across all participants learning rate parameters are very small, and reward sensitivity parameters are very large; this suggests that most of the learning occurred only after rewarded trials. The distribution of individual parameter estimates is shown in figure 20.

Table 13: Group-level parameter. Estimates are the means of posterior distribution and intervals are Bayesian HPDI credible interval. Note that following the pre-registration, in this model group-level differences were estimated explicitly only for the reward sensitivity parameters ρ.

	Non-refugees			Refugees			Group differences		
parameter	estimate	SE	95% CI	estimate	SE	95% CI	difference	diff. SE	diff. 95% CI
γ	0.71	0.06	(0.61, 0.82)	0.71	0.06	(0.61, 0.82)			
ρ	57.75	114.65	(0.98, 121.83)	85.78	181.28	(1.13, 178.93)	28.03	88.63	(-25.8, 76.83)
$\epsilon \times 10^4$	6.28	9.40	(0.02, 14.72)	6.28	9.40	(0.02, 14.72)			

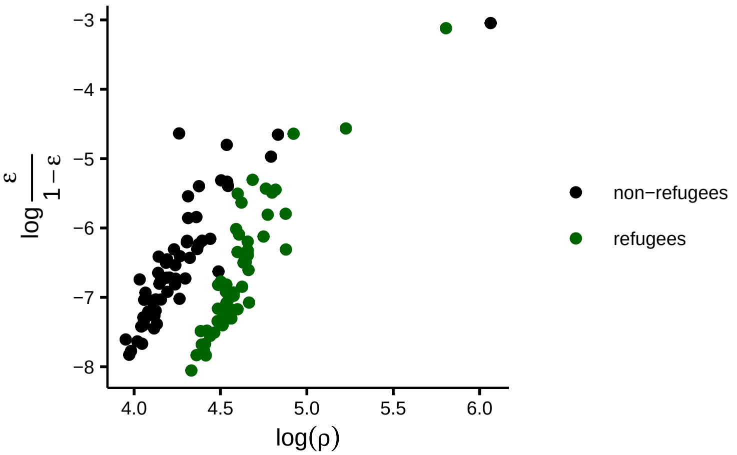

Figure 20: Relationship between learning rate (y axis) and reward sensitivity (x axis).

5 Proxy measures of socioeconomic status

Table 14: Combined results from Study 1 and Study 2 and 3. Group differences have been tested using t-tests, and p-values are not adjusted for multiplicity. Bayes factors (BF_{01}) represent evidence in favor of the null hypothesis and were calculated using the JZS prior (Rouder et al., 2009).

Study	Variable	t-statistic	p	Mean Non-Refugees	Mean Refugees	BF_{01}
**1**	Dependency Ratio	-0.406	0.686	0.284	0.310	5.639
	Bedroom per Person	-0.410	0.683	0.401	0.414	5.631
	People in Household	1.784	0.078	6.405	5.500	1.414
**2, 3**	People in Household	0.473	0.637	6.237	6.098	6.479
	Bedrooms in House	0.993	0.322	2.413	2.275	4.514
	Employed People in Household	1.733	0.086	1.388	1.196	1.752

References

1. Manning, C., Kilner, J., Neil, L., Karaminis, T., & Pellicano, E. (2016). Children on the autism spectrum update their behaviour in response to a volatile environment. *Developmental Science*, *20*(5). https://doi.org/10.1111/desc.12435
2. Pessiglione, M., Seymour, B., Flandin, G., Dolan, R. J., & Frith, C. D. (2006). Dopamine-dependent prediction errors underpin reward-seeking behaviour in humans. *Nature*, *442*(7106), 1042–1045. https://doi.org/10.1038/nature05051
3. Lewandowski, D., Kurowicka, D., & Joe, H. (2009). Generating random correlation matrices based on vines and extended onion method. *Journal of Multivariate Analysis*, *100*(9), 1989–2001. https://doi.org/10.1016/j.jmva.2009.04.008
4. Carpenter, B., Gelman, A., Hoffman, M. D., Lee, D., Goodrich, B., Betancourt, M., Brubaker, M., Guo, J., Li, P., & Riddell, A. (2017). Stan : A Probabilistic Programming Language. *Journal of Statistical Software*, *76*(1). https://doi.org/10.18637/jss.v076.i01
5. Gelman, A., & Rubin, D. B. (1992). Inference from Iterative Simulation Using Multiple Sequences. *Statistical Science*, *7*(4), 457–472. https://doi.org/10.1214/ss/1177011136
6. Vehtari, A., Gelman, A., & Gabry, J. (2017). Practical Bayesian model evaluation using leave-one-out cross-validation and WAIC. *Statistics and Computing*, *27*(5), 1413–1432. https://doi.org/10.1007/s11222-016-9696-4
7. Constantino, S. M., & Daw, N. D. (2015). Learning the opportunity cost of time in a patch-foraging task. *Cognitive, Affective, &Amp; Behavioral Neuroscience*, *15*(4), 837–853. https://doi.org/10.3758/s13415-015-0350-y
8. Perrin, S., Meiser-Stedman, R., & Smith, P. (2005). The children's revised impact of event scale (CRIES): Validity as a screening instrument for PTSD. *Behavioural and Cognitive Psychotherapy*, *33*(4), 487–498. https://doi.org/10.1017/s1352465805002419
9. Mahfoud, Z., Abdulrahim, S., Taha, M. B., Harpham, T., El Hajj, T., Makhoul, J., Nakkash, R., Kanj, M., & Afifi, R. (2011). Validation of the arab youth mental health scale as a screening tool for depression/anxiety in lebanese children. *Child and Adolescent Psychiatry and Mental Health*, *5*(1). https://doi.org/10.1186/1753-2000-5-9
10. Ziadni, M. (2011). Sources of human insecurity in post-war situations. *Journal of Human Security*, *7*(3), 23–36. https://doi.org/10.3316/jhs0703023
11. Panter-Brick, C., Eggerman, M., Gonzalez, V., & Safdar, S. (2009). Violence, suffering, and mental health in afghanistan: A school-based survey. *The Lancet*, *374*(9692), 807–816. https://doi.org/10.1016/s0140-6736(09)61080-1
12. Jellinek, M. S., Murphy, J. M., Little, M., Pagano, M. E., Comer, D. M., & Kelleher, K. J. (1999). Use of the pediatric symptom checklist to screen for psychosocial problems in pediatric primary care: A national feasibility study. *Archives of Pediatrics &Amp; Adolescent Medicine*, *153*(3). https://doi.org/10.1001/archpedi.153.3.254
13. Pizzagalli, D. A., Jahn, A. L., & O'Shea, J. P. (2005). Toward an objective characterization of an anhedonic phenotype: A signal-detection approach. *Biological Psychiatry*, *57*(4), 319–327. https://doi.org/10.1016/j.biopsych.2004.11.026
14. Furukawa, E., Shimabukuro, S., Alsop, B., & Tripp, G. (2017). Behavioral sensitivity of japanese children with and without ADHD to changing reinforcer availability: An experimental study using signal detection methodology. *Behavioral and Brain Functions*, *13*(1). https://doi.org/10.1186/s12993-017-0131-6
15. Huys, Q. J., Pizzagalli, D. A., Bogdan, R., & Dayan, P. (2013). Mapping anhedonia onto reinforcement learning: A behavioural meta-analysis. *Biology of Mood & Anxiety Disorders*,

3(1), 12. https://doi.org/10.1186/2045-5380-3-12
16. Rouder, J. N., Speckman, P. L., Sun, D., Morey, R. D., & Iverson, G. (2009). Bayesian t tests for accepting and rejecting the null hypothesis. *Psychonomic Bulletin & Review*, *16*(2), 225–237. https://doi.org/10.3758/PBR.16.2.225
